# Serum microRNA profile of rhesus macaques following ionizing radiation exposure and treatment with a medical countermeasure, Ex-Rad

**DOI:** 10.1038/s41598-024-54997-8

**Published:** 2024-02-24

**Authors:** Eric Russ, Oluseyi O. Fatanmi, Stephen Y. Wise, Alana D. Carpenter, Manoj Maniar, Sergey Iordanskiy, Vijay K. Singh

**Affiliations:** 1 Division of Radioprotectants, Department of Pharmacology & Molecular Therapeutics, Uniformed Services University of the Health Sciences, Bethesda, MD 20814 USA; 2 The Henry M. Jackson Foundation for the Advancement of Military Medicine, Bethesda, MD 20817 USA; 3 Graduate Program of Cellular and Molecular Biology, Uniformed Services University of the Health Sciences, Bethesda, MD 20814 USA; 4 Armed Forces Radiobiology Research Institute, Uniformed Services University of the Health Sciences, Bethesda, MD 20814 USA; 5 Onconova Therapeutics, Inc., Newtown, PA 18940 USA; 6 Division of Radioprotectants, Department of Pharmacology and Molecular Therapeutics, F. Edward Hébert School of Medicine, 4301 Jones Bridge Road, Bethesda, MD 20814-2712 USA; 7 Present Address: Palm Pharmaceuticals, Inc, 46750 Sentinel Drive, Fremont, CA 94539 USA

**Keywords:** Ex-Rad, miRNA, Rhesus macaque, Total-body irradiation, Radiation countermeasure, ON01210, RNA, Molecular biology

## Abstract

Exposure to ionizing radiation (IR) presents a formidable clinical challenge. Total-body or significant partial-body exposure at a high dose and dose rate leads to acute radiation syndrome (ARS), the complex pathologic effects that arise following IR exposure over a short period of time. Early and accurate diagnosis of ARS is critical for assessing the exposure dose and determining the proper treatment. Serum microRNAs (miRNAs) may effectively predict the impact of irradiation and assess cell viability/senescence changes and inflammation. We used a nonhuman primate (NHP) model—rhesus macaques (*Macaca mulatta*)—to identify the serum miRNA landscape 96 h prior to and following 7.2 Gy total-body irradiation (TBI) at four timepoints: 24, 36, 48, and 96 h. To assess whether the miRNA profile reflects the therapeutic effect of a small molecule ON01210, commonly known as Ex-Rad, that has demonstrated radioprotective efficacy in a rodent model, we administered Ex-Rad at two different schedules of NHPs; either 36 and 48 h post-irradiation or 48 and 60 h post-irradiation. Results of this study corroborated our previous findings obtained using a qPCR array for several miRNAs and their modulation in response to irradiation: some miRNAs demonstrated a temporary increased serum concentration within the first 24–36 h (miR-375, miR-185-5p), whereas others displayed either a prolonged decline (miR-423-5p) or a long-term increase (miR-30a-5p, miR-27b-3p). In agreement with these time-dependent changes, hierarchical clustering of differentially expressed miRNAs showed that the profiles of the top six miRNA that most strongly correlated with radiation exposure were inconsistent between the 24 and 96 h timepoints following exposure, suggesting that different biodosimetry miRNA markers might be required depending on the time that has elapsed. Finally, Ex-Rad treatment restored the level of several miRNAs whose expression was significantly changed after radiation exposure, including miR-16-2, an miRNA previously associated with radiation survival. Taken together, our findings support the use of miRNA expression as an indicator of radiation exposure and the use of Ex-Rad as a potential radioprotectant.

## Introduction

Radiological and nuclear emergencies pose a significant threat to public health. According to the current standards set by the Centers for Disease Control and Prevention, acute radiation syndrome (ARS) is a general term for the health effects that arise following whole-body or significant partial-body exposure to > 0.7 Gy in a short period of time^1^. Traditionally, ARS is divided into three main subsyndromes: hematopoietic, gastrointestinal, and neurovascular. The first and only treatable subsyndrome, hematopoietic-ARS (H-ARS), is primarily characterized by the rapid death of circulating blood cells and the destruction of bone marrow. This results in myelosuppression and may lead to an opportunistic infection or hemorrhage, resulting in death^1,2^. Moreover, radiation exposure has long been associated with the development of chronic inflammation and fibrosis^3–5^. Counteracting these effects of radiation exposure is critical for civilian populations as well as military servicemembers against radiological and nuclear events^6–8^.

Previously, we explored the microRNA (miRNA) landscape in nonhuman primates (NHPs) prior to and following radiation exposure via a qPCR array^9^. miRNAs are small non-coding RNAs of 19–25 nucleotides in length and are known to play a role in every major biological process^10,11^. NHP models are incredibly valuable resource that offers a higher degree of conservativity to humans than rodent and porcine models^12,13^. However, the cost of NHPs and the limited number of facilities that support their use make NHP data valuable. Therefore, in order to validate our previous findings, expand our vision of miRNA spectra, and to enhance understanding of the changes of miRNA in response to radiation exposure, the first objective of this study was to repeat our previous report using a more powerful approach, miRNA-sequencing.

ON01210 ((E)-4-carboxystyrl-4-chlorbenzysulfone), more commonly known as Ex-Rad, is potential radiation medical countermeasure (MCM) under development. Ex-Rad was originally identified as a drug of interest during a compound library screening experiment where it exhibited radioprotectant properties^14^. It was later found that subcutaneous (*sc*) and oral prophylactic administration of Ex-Rad protected against H-ARS as well as gastrointestinal acute radiation syndrome (GI-ARS), and produced a significant survival advantage against radiation exposure in mice^15–17^. Additional analysis revealed that Ex-Rad prevents radiation-induced apoptosis through the activation of the PI3-Kinase/AKT pathways^18^. Of note, this effect of Ex-Rad prolongs white blood cell and endothelial cell survival in response to radiation exposure. Using an NHP model, we have recently demonstrated partial restoration of metabolites and proteins by Ex-Rad treatment which were altered as a result of ionizing radiation exposure^19,20^. Currently, Ex-Rad is considered to be a promising MCM for H-ARS and has completed a phase I clinical trial that demonstrated its safety, tolerability and pharmacokinetics in humans^21^. To gain a deeper understanding of Ex-Rad and its impact on the serum miRNA profile, the second objective of this study was to examine the effect of Ex-Rad administration on miRNA following radiation exposure in NHPs.

To examine the effect of Ex-Rad on NHPs exposed to radiation, a group of 10 NHPs were administered Ex-Rad 36 and 48 h post-irradiation. Another group of NHPs were administered Ex-Rad at 48 and 60 h post-irradiation. These two regimens were termed Ex-Rad I and Ex-Rad II, respectively. To monitor the serum miRNA profile following a single dose of 7.2 Gy gamma-irradiation, blood samples were collected from rhesus macaques 96 h prior to irradiation and at four timepoints post-irradiation (24, 36, 48, and 96 h). Following blood sample collection, serum was separated and miRNA was isolated for miRNA-sequencing analysis. Our results corroborated our previous findings for several miRNAs and their modulation in response to radiation exposure. Additionally, we observed that Ex-Rad treatment partially restored the serum miRNA profile to pre-irradiation levels compared to untreated and irradiated NHPs. In brief, these findings support the use of miRNA expression as an indicator of radiation exposure and the use of Ex-Rad as a potential MCM.
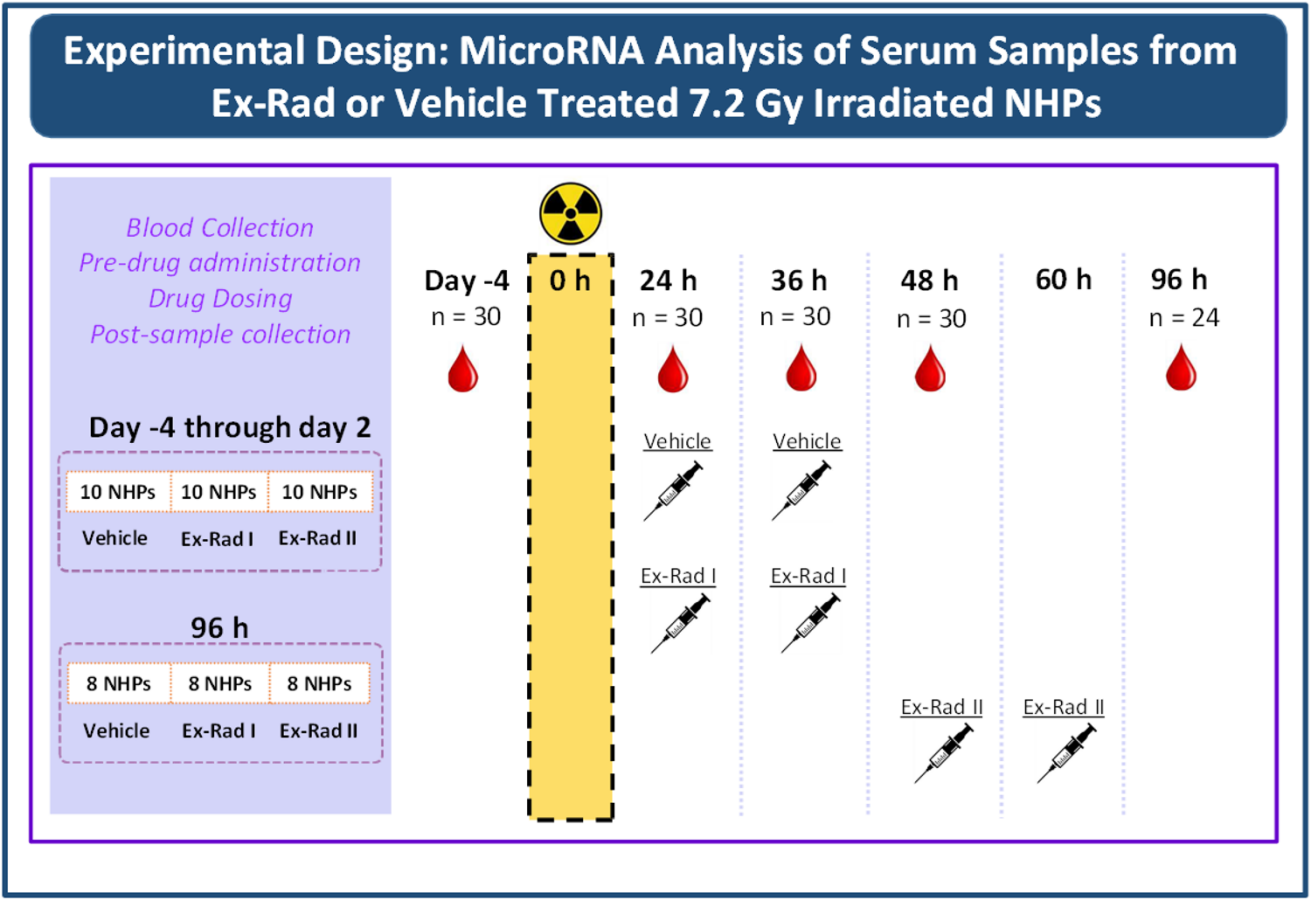


## Results

### Changes in the serum miRNA profile of rhesus macaques following radiation exposure

**Figure 1:**
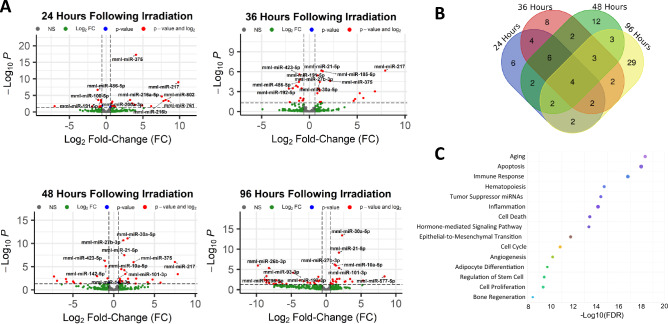
Changes in the serum miRNA profile of rhesus macaques following radiation exposure. (**A**) Volcano plots displaying significantly modulated miRNAs following radiation exposure at each time point. Grey dots indicate non-significantly changed miRNAs (NS), green dots indicate > 1.5-fold changes in serum miRNA in irradiated vs non-irradiated animals, and red dots indicate significantly changed miRNAs (p < 0.05). (**B**) Venn diagram depicting uniquely and shared miRNAs for each time point after irradiation. The area above the horizontal dash line indicates p < 0.05. (**C**) Key biological processes associated with the significantly modulated miRNAs 24 h post-irradiation. The horizontal axis indicates type I error: − Log10 False Discovery Rate adjusted p-value.

To determine if radiation exposure modulates the serum miRNA profile of rhesus macaques, we collected blood samples 96 h prior to TBI as a baseline and 24, 36, 48, and 96 h following TBI with a single dose of 7.2 Gy gamma-radiation. Due to the use of a MCM under development to two groups of animals, the number of samples for this analysis decreases from n = 30 (96 h pre-irradiation and 24 h post-irradiation) to n = 20 (36 and 48 h post-irradiation) and n = 8 (96 h post-irradiation as stated under blood collection subsection under “Materials and methods” section). A total of 400 miRNA were detectably expressed following edgeR’s standard filtering process (Supplemental Table 1)^22^. For each timepoint following irradiation, we identified significantly altered miRNAs, defined by a fold-change of > 1.5 and a false discovery rate adjusted p-value of < 0.05, through edgeR’s differential expression analysis that implements a quasi-likelihood negative binomial generalized log-linear model (GLM) function to normalize the data (Fig. 1A). Across all of the timepoints, there does not appear to be a clear pattern of miRNA upregulation versus downregulation (Supplemental Table 2). Among the significantly altered miRNAs, there is a high degree of overlap between the timepoints, aside from 96 h post-irradiation. Unlike the other timepoints, which contain fewer uniquely modulated miRNAs than the amount they share with the other timepoints, the majority of altered miRNAs at 96 h post-irradiation are unique to this timepoint (Fig. 1B).

To determine which biological processes the identified modulated miRNAs are linked to, we used the published tool TAM 2.0 to analyze our dataset^23^. This tool was created through the curation of over 9000 papers to create reference miRNA sets that can be linked to general functions. Although this is not a precise analysis, as miRNAs can have multiple targets and functions depending on the cell type, it can be used to identify any major discrepancies between our significantly modulated miRNAs and the functions that are expected to be activated in response to irradiation^24^. In general, the identified biological processes align with processes that may be modulated in response to irradiation, including: apoptosis, immune response, hematopoiesis, inflammation, cell cycle, and bone regeneration (Fig. 1C). According to publications where the effect of similar radiation doses was analyzed using different methods and targets, such as mRNA or protein analysis, the same pathways were normally changed upon irradiation^25–29^. Interestingly, miRNAs involved in the aging-related pathways were found to be highly enriched. This is consistent with recent publications indicating that aging-related changes in cells (senescence) are associated with oxidative stress and elevated inflammation^30–32^. An example of an age-related miRNA that is upregulated following radiation exposure is miR-7. This miRNA is elevated in old fibroblasts compared to young fibroblasts and binds to the 3′ UTR of epidermal growth factor receptor (EGFR), leading to an age-related loss of fibroblast to myofibroblast differentiation^33^. Additionally, miR-7 is suggested to regulate cell stemness and radioresistance through targeting krüppel-like factor 4 (KLF4), a well-known stemness-associated transcription factor^34^. In our analysis, the level of miR-7 was twofold higher in the serum of NHPs 24 h following radiation exposure and may contribute to the characteristic loss of normal stem cell function that occurs during ARS and the subsequent lack of efficient tissue renewal. A full list of the miRNAs that TAM 2.0 associated with each pathway is provided in Supplemental Table 3. Notably, mature miRNAs with the same sequence may arise from distinct genomic locations (paralogs). In our analysis, we focused on mature miRNA sequences only, but TAM 2.0 backtraces the mature miRNA into every potential pri-miRNA which may have produced it. These paralogous pri-miRNAs are denoted by the addition of “− 1”, “− 2”, etc. depending on how many exist for the same mature miRNA sequence.

### Hierarchical clustering of NHPs based on their miRNA profile

**Figure 2:**
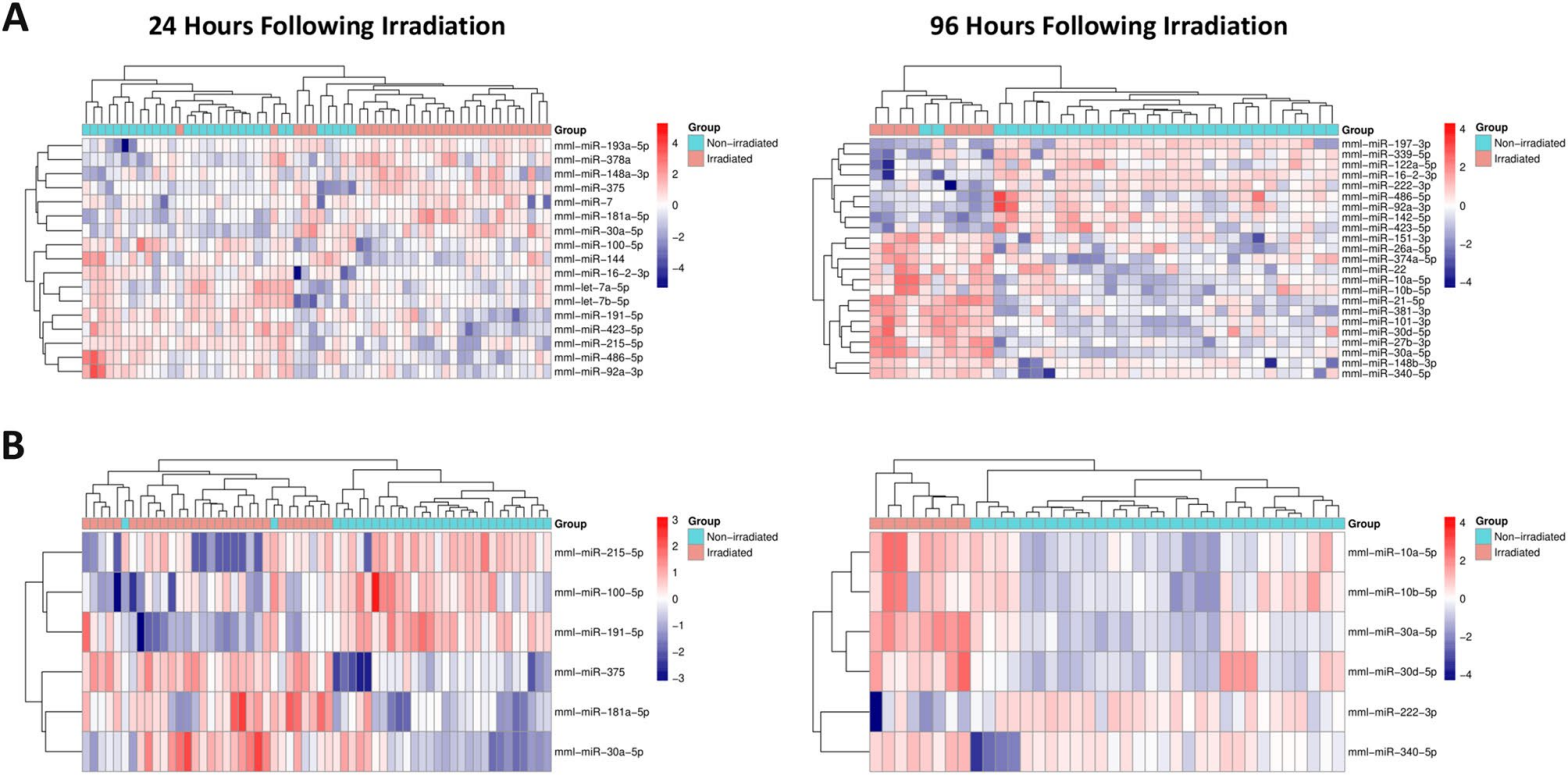
Hierarchical clustering of NHPs based on their miRNA profile. (**A**) Heatmap depicting hierarchical clustering of NHPs based on significantly modulated miRNAs in the serum samples from unirradiated and 7.2 Gy-irradiated animals, harvested at 24 h (left) and 96 h (right) post-exposure. (**B**) Heatmap and hierarchical clustering with the 6 miRNA that had the highest degree of correlation with irradiation for each time point, unirradiated and 7.2 Gy-irradiated animals, harvested at 24 h (left) and 96 h (right) post-exposure.

To assess if the groups of unirradiated and irradiated NHPs can be efficiently separated from each other based on their serum miRNA profile, we performed hierarchical clustering. Only significantly differentially expressed miRNAs that were detectably expressed in at least half of the samples were chosen for analysis. Using this criterion, although the expression of each miRNA is not entirely consistent within the unirradiated and irradiated groups, the samples were efficiently separated into two main clusters at 24 and 96 h post-irradiation (Fig. 2A). If miRNA expression is to be used as a biodosimetry tool for radiation exposure, it is critical to identify a limited number of miRNAs within the pool that display significantly differential expression. Therefore, we performed the hierarchical clustering with six miRNAs per timepoint to demonstrate that it is possible to efficiently identify unirradiated versus irradiated NHPs with this limited number of miRNA (Fig. 2B). A similar analysis for the other timepoints is shown in Supplemental Fig. 1. Of note, among the miRNAs within the groups of six potential biomarkers, none were consistent between the 24 and 96 h profiles, suggesting that different biodosimetry markers might be needed for different time points after radiation exposure.

### Trends in serum miRNA levels across time following irradiation

**Figure 3:**
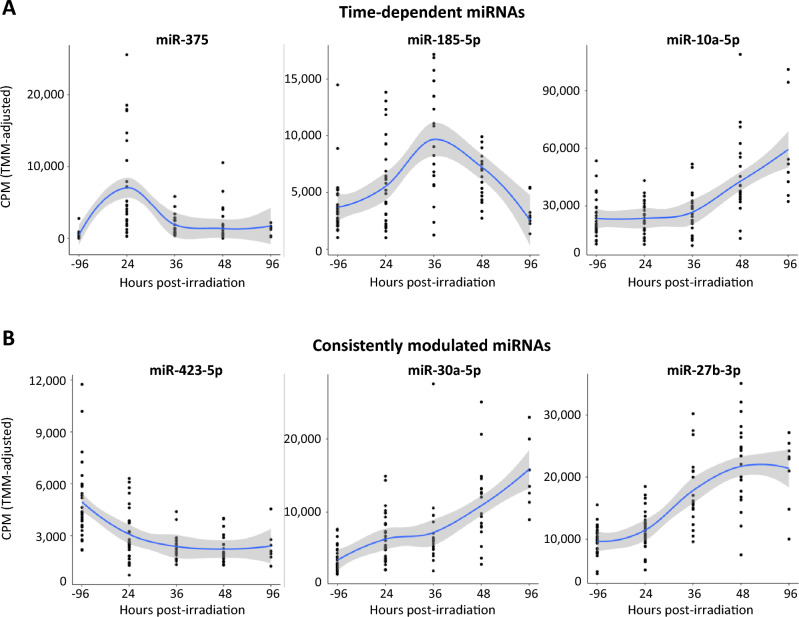
Trends in serum miRNA levels across time following radiation exposure. Scatterplots with locally estimated scatterplot smoothing (LOESS) to illustrate the trends in serum miRNA modulation over time following irradiation. Different trends can be observed, with some miRNAs (**A**) following a time-dependent modulation and other miRNAs (**B**) experiencing a consistent modulation for at least 96 h after radiation exposure. In all plots: *CPM* count per million values.

To understand the patterns of serum miRNA modulation over time that can emerge following irradiation, we generated locally estimated scatterplot smoothing (LOESS) plots. For this analysis, we only visualized miRNAs that were expressed in over 90% of the samples (for all included miRNAs and their corresponding plots, reference Supplemental Figs. 2–4). As shown in Fig. 3A, the effect of irradiation on the serum concentration of a specific miRNA can be an early response (24–36 h) and slow return to baseline (miR-375 and miR-185-5p, respectively), or delayed and not visible until 96 h post-irradiation (miR-10a-5p). Other miRNAs demonstrated a different pattern of response to radiation: either a prolonged decline (miR-423-5p) or a long-term increase in their serum concentration (miR-30a-5p, miR-27b-3p) (Fig. 3B).

### Ex-Rad treatment partially restores the serum miRNA profile to pre-irradiation levels compared to untreated irradiated animals

**Figure 4:**
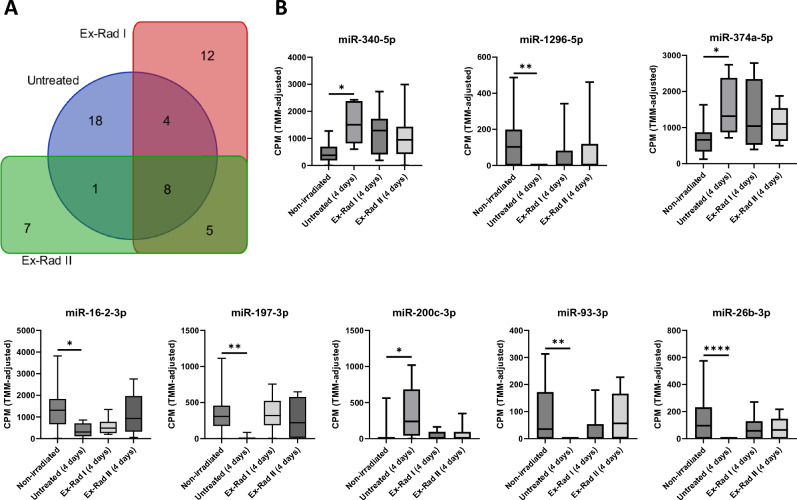
Ex-Rad treatment partially restores the serum miRNA profile to pre-irradiation levels compared to control. (**A**) Venn diagram depicting shared and uniquely modulated miRNAs for each treatment regimen (untreated, Ex-Rad I, and Ex-Rad II) 96 h post-irradiation. (**B**) Expression of miRNAs that were significantly modulated in untreated NHPs and restored to insignificant levels in Ex-Rad treated NHPs 96 h post-irradiation (**B**). *CPM* count per million values, calculated separately for each miRNA. *p < 0.05, **p < 0.01, ***p < 0.001, ****p < 0.0001.

To assess the potential effect of Ex-Rad treatment on the serum miRNA profile, we identified miRNAs that were significantly modulated 96 h post-irradiation in untreated and Ex-Rad-treated NHPs in the groups Ex-Rad I and Ex-Rad II compared to unirradiated NHPs. Overall, we found that 31 miRNAs were significantly modulated in untreated NHPs, 29 miRNAs were significantly modulated in NHPs of Ex-Rad I group, and 21 miRNAs were significantly modulated in NHPs under the Ex-Rad II group (Fig. 4A). Among the 55 modulated miRNAs, 18 were specific to the untreated NHPs, 12 to the Ex-Rad I NHPs, and 7 to the Ex-Rad II NHPs. In total, eight notable including miRNAs, miR-340-5p, miR-1296-5p, miR-374a-5p, miR-16-2-3p, miR-197-3p, miR-200c-3p, miR-93-3p, and miR-26b-3p were found to be restored to pre-irradiation levels in response to both Ex-Rad treatments. The quantitative ratios of these miRNAs in the serum of irradiated and Ex-Rad treated animals versus unirradiated controls are shown in Fig. 4B.

## Discussion

In the present study, we assessed the serum miRNA profile of NHPs following irradiation and examined the effect of Ex-Rad treatment on the serum miRNA profile of irradiated NHPs. This was performed by collecting blood samples from NHPs at various timepoints pre- and post-irradiation and/or drug treatment, followed by serum separation, miRNA isolation, and miRNA sequencing. Although it is known that radiation exposure modulates the expression of various miRNAs, how the serum miRNA landscape evolves over time has not been clearly identified. Moreover, NHP and human data are limited in comparison to rodent and porcine data, and the effect of Ex-Rad treatment on the serum miRNA profile has yet to be studied.

The results of this study corroborate previous findings that the serum miRNA profile is altered in response to radiation. Four miRNAs were significantly modulated in every timepoint: miR-216a-5p, miR-423-5p, miR-30a-5p, and miR-577-5p. All except for miR-577-5p were previously shown to be modulated in response to irradiation, including in our previous analysis of NHPs that implemented a qPCR panel to quantify miRNA expression^9,35–37^. In a comparison with inflammatory conditions (viral infection, bacterial infection, and inflammatory bowel disease), the pattern of modulation for each of the four miRNAs appears to be unique to radiation exposure (Supplemental Table 4). However, while miR-577-5p was only found in the serum in irradiated NHPs, it may not be a suitable biomarker as only a small portion of radiation exposed NHPs had a detectable level. Interestingly, miR-423-5p was elevated in the serum of patients with hepatitis C virus (HCV) infection, pulmonary tuberculosis or inflammatory bowel disease, but consistently lowered in the serum of NHPs following irradiation in our study. While the cause of miR-423-5’s expression pattern in response to radiation compared to other inflammatory conditions is unknown, it is worth noting that miR-423-5p expression negatively correlates with radiosensitivity in colorectal cancer cells and that knockdown of miR-423-5p alone can provide a significant radioprotective effect^38^. These results may suggest that miR-423-5p downregulation is an intrinsic response to protect against radiation-induced cell death, but more work is needed to support this.

An miRNA-based biodosimetry panel would require a combination of multiple miRNAs with distinct characteristics to overcome the underlying variability of serum analysis in a clinical setting. Since miR-423-5p’s modulation pattern appears to be unique to irradiation, is relatively consistent across the NHP samples, and remains repressed in the serum for a prolonged period of time (at least 96 h), this type of miRNA may be suitable to include in a panel for the detection of radiation exposure. In our previous study, we identified a five-miRNA signature that could accurately distinguish between unirradiated and irradiated NHPs. The current sequencing-based analysis specifies that three out of the five miRNAs are significantly modulated in a similar manner: miR-375, miR-215-5p, and miR-30a-5p (Supplemental Fig. 4). Additional miRNAs were also significantly modulated in a similar manner between our previous study and this study; miR-10b-5p, miR-150-5p, miR-16-2-3p, and miR-100-5p. There are other studies where the importance of miR-150 has been discussed for developing radiation biodosimetry^39,40^. Some miRNAs were not in agreement between our previous study and the current study, which can be expected as there are inherent variances that may arise due to the different methodologies of quantifying miRNA expression (qPCR array versus miRNA-seq) or different experimental conditions, such as dose of radiation or cohorts of animals.

While the precise function of individual miRNAs is dependent on the context, several radiation-modulated miRNAs are associated with organ failure and may be useful in future studies that assess indicators of ARS. For instance, miR-30a-5p was found to inhibit osteogenesis through targeting the runt-related transcription factor 2 (RUNX2), a key regulator of bone marrow mesenchymal stem cell differentiation into the osteogenic lineage^41–43^. If circulating miR-30a-5p is increased following irradiation as our data suggests, it could contribute to radiation-induced degradation of the bone marrow and result in hematopoietic dysfunction.

It is also important to note that while these types of large-scale analyses are beneficial for identifying potential miRNAs of interest, the sensitivity of qPCR arrays and miRNA-seq approaches may not be as efficient as a separate qPCR analysis for each individual miRNA. Due to the limited availability of NHP samples, it was not feasible to pair miRNA-seq with individual qPCR experiments herein, but future studies may take advantage of our current and previously identified miRNAs to conduct a more stringent analysis.

The ability of MCMs to restore or protect against changes to the serum miRNA following irradiation has been shown previously. Our earlier study demonstrated that gamma-tocotrienol, another promising MCM under advanced development, restored gamma-irradiation-induced alteration of a few miRNAs: miR-30a-5p, miR-126-5p and miR-375^44,45^. It is important to note that these three miRNAs have also been shown to be altered in response to radiation exposure in the current study. There are several reports where other promising MCMs were shown to restore various miRNAs altered by radiation exposure, although these studies were conducted in a murine model of H-ARS. In all these studies, cobalt-60 gamma-radiation has been used to irradiate CD2F1 mice^46–48^. These MCMs are CDX-301 (recombinant human protein of the Fms-related tyrosine kinase 3 ligand)^46^, gamma-tocotrienol^47^, and delta-tocotrienol^48^. CDX-301 has been shown to restore the levels of large number of miRNAs in mice exposed to 7 Gy radiation, a non-lethal dose of radiation. The GT3 murine model study was also conducted with sublethal dose of radiation while the delta-tocotrienol study has been accomplished with a lethal dose of radiation. In line with these previous studies, our current study suggests that administration of Ex-Rad following radiation exposure may partially alleviate radiation-induced changes to the serum miRNA profile. Compared to untreated irradiated NHPs, Ex-Rad administration reduces the overall number of significantly altered miRNAs 96 h post-irradiation with the unirradiated NHP sample set as the baseline (Supplemental Table 5). Some notable examples include miR-197-3p, miR-200c-3p, and miR-16-2-3p. When comparing the untreated and Ex-Rad treated samples directly against each other, it is observed that four miRNAs are significantly modulated between the untreated NHPs and Ex-Rad treated NHPs, with both Ex-Rad treatment schedules displaying the same four significantly modulated miRNAs: miR-7174-3p, miR-26b-3p, miR-144, and miR-93-3p (Supplemental Table 6).

In Ex-Rad treated NHPs, the expression of miR-197-3p recovered to baseline levels following 96 h post-irradiation whereas untreated NHPs had a significant drop in serum miRNA concentration. Downregulation of miR-197 is associated with various inflammatory pathologies. Previously, miR-197 was found to be downregulated in patients suffering from psoriasis, a chronic inflammatory skin disorder^49,50^. Upon molecular analysis, miR-197 was shown to be involved in a positive–negative feedback loop with interleukin 17A (IL-17A) signaling^51^. In this feedback loop, IL-17A signaling induces miR-197 expression and miR-197 binds to the 3’ UTR region of the receptor for IL-17A, IL-17RA, thereby attenuating the signaling pathway. This downregulation was suggested to contribute to the pro-inflammatory environment of psoriasis due to excess IL-17A signaling. Additionally, a similar positive–negative feedback loop was found between IL-22, a member of the IL-10 family that has both pro- and anti-inflammatory properties, and miR-197 by the same authors^52^. Earlier, it has been shown that IL-22 expression is upregulated by ionizing radiation exposure and this cytokine, in turn, modulates both acute and chronic inflammatory responses in the intestine^53,54^. In another disease context, patients suffering from familial Mediterranean fever (FMF), an inherited autoinflammatory disease caused by mutations in the Mediterranean Fever (MEFV) gene, serum miR-197-3p concentration was significantly decreased compared to healthy individuals^55^. Notably, this decrease in serum miR-197-3p was significantly more noticeable in severe patients than mild ones. To uncover the potential role of miR-197-3p, the authors examined inflammatory pathway activation following transfection of miR-197-3p into synovial fibroblasts, monocytes, and macrophages. Importantly, miR-197-3p was found to have an anti-inflammatory effect in multiple cell types via inhibition of IL-1B expression and secretion, potentially through downregulation of the IL-1B receptor, IL-1R1. Overall, these earlier studies suggest that low levels of serum miR-197-3p may either indicate or contribute to a pro-inflammatory state.

The miRNA miR-200c-3p displayed an opposite trend and was significantly elevated in untreated NHPs 96 h post-irradiation. Under stress conditions, cardiomyocytes upregulate and secrete miR-200c-3p^56^. It was found that exosomal transfer of this miRNA from cardiomyocytes to epithelial cells leads to inhibited angiogenesis via reduced proliferation, migration, and tubule formation. Additionally, mice treated with an antagomir against miR-200c-3p during transverse aortic constriction (TAC)-induced cardiac pressure overload experienced a milder hypertrophic phenotype (smaller fibrotic areas, a higher abundance of capillaries, and a more preserved cardiac ejection fraction) compared to untreated mice. Another study indicates that miR-200c is overexpressed in the normal human keratinocytes after irradiation, facilitates their senescence phenotype and overall promotes the development of radiation-induced oral mucositis^57^. Together, this data suggests that miR-200c-3p is both a marker and a contributor of heart-related injuries.

Of most interest is miR-16-2, which we previously showed to negatively correlate with rhesus macaque survival following 7.2 Gy radiation^9^. While untreated NHPs experienced a significant decrease in the serum concentration of miR-16-2 following irradiation, Ex-Rad treated NHPs are insignificantly altered compared to pre-irradiation samples. This may suggest that Ex-Rad treated NHPs have a higher likelihood of survival.

Though there are several studies with miRNAs using irradiated NHPs (rhesus macaques or baboons), results of these studies are not directly comparable due to different experimental conditions in respect of animal species, radiation source, quality^58,59^, dose, dose rate, exposure type (total-body or partial-body), timepoint for sample collection (specifically during ARS or DEARE (delayed effects of acute radiation exposure) timeframe), or levels of supportive care provided to experimental animals, etc.^60–63^.

While a major limitation to our study is our incomplete understanding of the involvement of various miRNAs in radiation pathogenesis and their complex nature, the results are in agreement with our recent studies that assessed the serum metabolic and serum proteomic profiles of NHPs following irradiation and Ex-Rad treatment^19,20^. In these studies, we observed higher levels of inflammation and oxidative stress in the untreated and irradiated NHPs, and a similar, partial restoration to pre-irradiation levels in response to Ex-Rad treatment.

## Materials and methods

### Animals

This study was performed using 30 male naïve rhesus macaques (*Macaca mulatta*, Chinese sub-strain) that were acquired from the National Institutes of Health Animal Center (NIHAC, Poolesville, Maryland). All of the NHPs were 4–5 years old and body weights ranged from 4.3 to 6.2 kg. This study was conducted at a vivarium accredited by the Association for Assessment and Accreditation of Laboratory Animal Care (AAALAC)-International. Prior to the initiation of the study, the animals underwent a quarantine period of six weeks. Additional details such as the animal feed, husbandry, health monitoring, environmental enrichment, and basic care utilized throughout the experimental period have been previously been discussed^19^. Due to a suppressed immune system following irradiation, single housing was preferred. Irradiated animals are more prone to infection and paired-housing was not practical during the experiment. Single housing also averts conflict injuries that often occur in paired-housing. Although not paired-housed, the animals were able to have limited interactions with neighboring animals placed in adjacent cages through the cage dividers^64^. All animal-based procedures were approved (Protocol # P2013-12-016 approved on March 12, 2014) by the Institutional Animal Care and Use Committee (IACUC, Armed Forces Radiobiology Research Institute) and second-tier approval was obtained by the Department of Defense Animal Care and Use Review Office (ACURO). All animal procedures were performed according to the *Guide for the Care and Use of Laboratory Animals* of the Institute of Laboratory Animal Resources, National Research Council, U.S. National Academy of Sciences^65^. This study is reported in accordance with ARRIVE guidelines.

### Ex-Rad administration

The details of the drug, Ex-Rad, including its preparation, concentration, administration, and other pertinent details have been described earlier^20^. To summarize, ten NHPs were randomly placed in one of the following three treatment regimens: Ex-Rad administered 24 and 36 h post-irradiation Ex-Rad I), Ex-Rad administered 48 and 60 h post-irradiation (Ex-Rad II), and vehicle administered 24 and 36 h post-irradiation. The total dose administered was 40 mg/kg; the actual injection volume was calculated based on the individual animal’s body weight at day 0 for each NHP. Approximately 48 h prior to the scheduled drug administration, the hair surrounding the injection site (dorsal scapular region—between the shoulder blades) was shaved to allow for easy visual monitoring of any adverse reaction such as a rash, inflammation, irritation, or abscess formation. Immediately before the drug or vehicle administration, the injection site was cleaned with 70% isopropyl alcohol and allowed to air dry. Ex-Rad or the vehicle was administered *sc* using a sterile 21–24-gauge needle attached to a 3–6 ml disposable luer-lock syringe.

### Irradiation

Dose rate measurements were performed prior to irradiation as described previously^66–68^. The irradiation procedure, including animal sedation, grouping, recovery, and monitoring are discussed earlier^19^. Briefly, the animals were irradiated in pairs based on their abdominal widths. To limit movement during the procedure, animals were sedated and placed in a plexiglass restraint box, which was then secured to the irradiation platform with ratcheting straps. For this study, animals received a precise total-body midline dose of 7.2 Gy ^60^Co gamma-radiation at a rate of 0.6 Gy/min^69^.

### Blood collection and serum separation

For blood sample collection, animals were removed from their cage via the pole-and-collar method, and secured in a custom-made restraint chair as mentioned earlier^20^. Once the blood was collected, it was placed in serum-separator tubes and allowed to clot for at least 30 min prior to being centrifuged (10 min, 400×*g*). The serum was transferred into empty cryotubes which were then stored at − 70 °C until use. For samples collected at 24 h post-irradiation, Ex-Rad was administered after the sample collection, prior to the first dose of Ex-Rad for the Ex-Rad I group. Similarly, for the samples collected at the 36 h timepoint, from this Ex-Rad-treated group, samples were taken just prior to the administration of the second dose of Ex-Rad. Lastly, samples collected at 48 h post-irradiation were collected just before the administration of the first dose of drug to the Ex-Rad II group. In brief, blood samples for serum collection were collected prior to drug administration at each time point, and samples were collected at 96 h pre-irradiation, and 24, 36, 48, and 96 h post-irradiation for miRNA analysis. Thus, although drug administrations occurred at 24 and 36 h for Ex-Rad I, and at 48 and 96 h for Ex-Rad II, the statistically significant effects of Ex-Rad I can only be studied at 48 and 96 h, while the effects of Ex-Rad II can only be viewed at 96 h when both doses of drug were administered to their respective groups.

### RNA extractions, library preparation and sequencing

Total RNA including miRNA was extracted from NHP serum samples using miRNeasy (QIAGEN). All extracted RNA was used in the library preparation following Illumina’s TruSeq -small-RNA-sample preparation protocols (Illumina, San Diego, CA, USA). Quality control analysis and quantification of the DNA library were performed using Agilent Technologies 2100 Bioanalyzer High Sensitivity DNA Chip. Single-end sequencing 50 bp was performed on Illumina’s Hiseq 2500 sequencing system following the manufacture’s recommended protocols.

### Bioinformatics analysis

Raw reads were subjected to an in-house program, ACGT101-miR (LC Sciences, Houston, Texas, USA) to remove adapter dimers, junk, low complexity, common RNA families (rRNA, tRNA, snRNA, snoRNA) and repeats. Sequences with a length of 18 ~ 26 nucleotides were mapped and quantified using the mapper and quantification functions of MiRDeep2 against the known Macaca mulatta (rhesus macaque) precursor sequences and mature miRNA sequences, obtained from the miRBase repository^70^. The resulting miRNA counts were normalized using edgeR to obtain CPM (TMM-adjusted) values^71^.

### Analysis of differential expressed miRNAs

Differential expression of miRNAs based on normalized deep-sequencing counts was determined by using edgeR’s built-in differential expression function to obtain the fold-changes, p-values, and false discovery rate (FDR) adjusted p-values for each comparison. The edgeR package implements a quasi-likelihood negative binomial generalized log-linear model (GLM) function to normalize the data. Significantly modulated miRNAs had a cutoff of > 1.5 fold-change and < 0.05 FDR adjust p-value, unless otherwise stated. For some comparisons, only miRNAs that were expressed in at least 50% of the samples were included, as stated.

### The prediction of modulated pathways

To determine which biological processes the identified modulated miRNAs are linked to, we used the published tool TAM 2.0 to analyze our dataset^23^. Although the precise function of an individual miRNA may be context dependent or relatively unknown compared to typical transcripts, this tool was created through the curation of over 9000 papers to create reference miRNA sets that can be linked to general functions.

### Supplementary Information

Supplementary Information.

## Data Availability

All data generated or analyzed during this study are included in this published article (and its Supplementary Information files).
