# Supplementary material for: Serum microRNA profile of rhesus macaques following ionizing radiation exposure and treatment with a medical countermeasure, Ex-Rad
Source: Sci Rep. 2024 Feb 24;14:4518. doi: 10.1038/s41598-024-54997-8 (PMC10894202; doi:10.1038/s41598-024-54997-8)
Supplement: Supplementary file 1 — Supplementary Information. [file 41598_2024_54997_MOESM1_ESM.pdf]

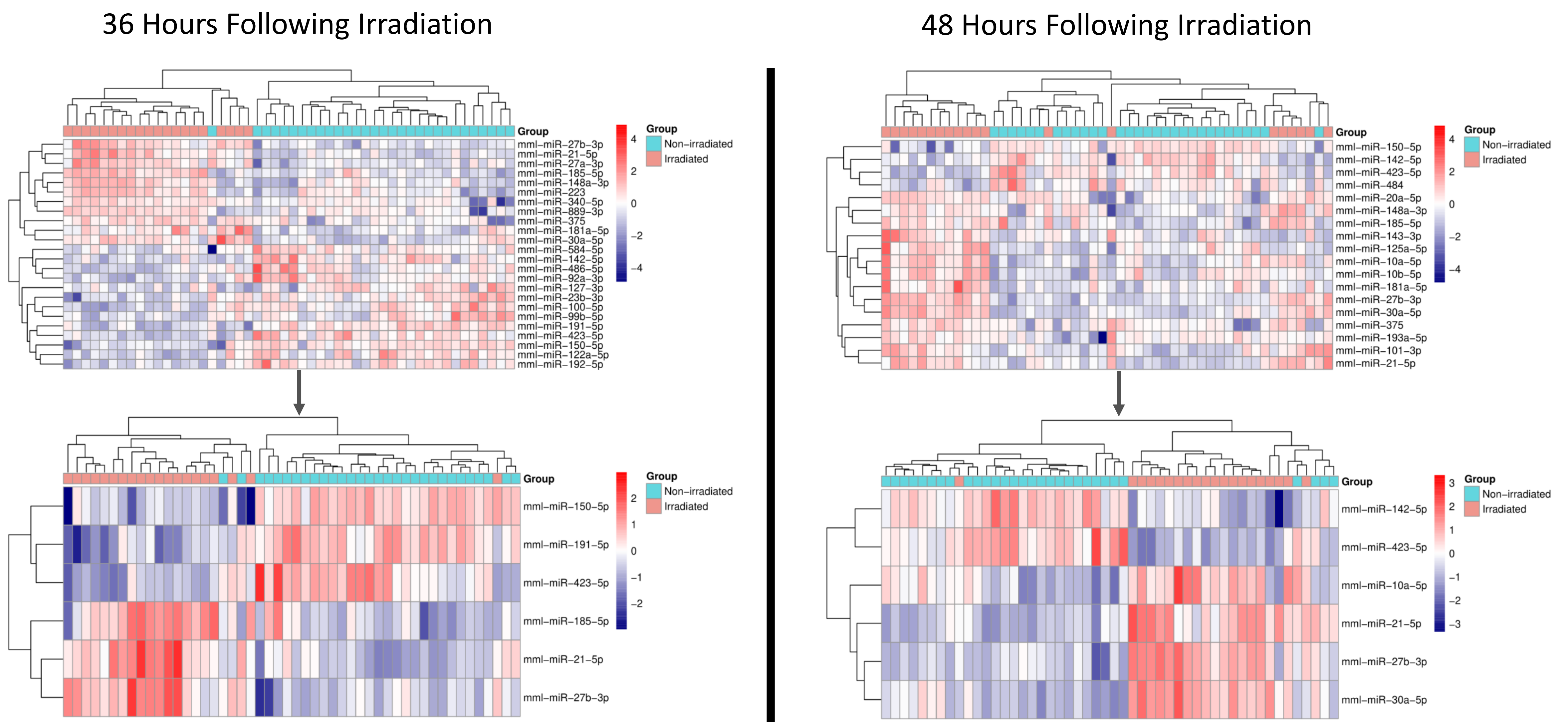

Supplemental Figure 1. Hierarchical clustering of significantly modulated miRNAs expressed in at least half of all samples (top) and the six most highly radiation-correlated miRNAs (bottom) 36 and 48 hours post-irradiation.

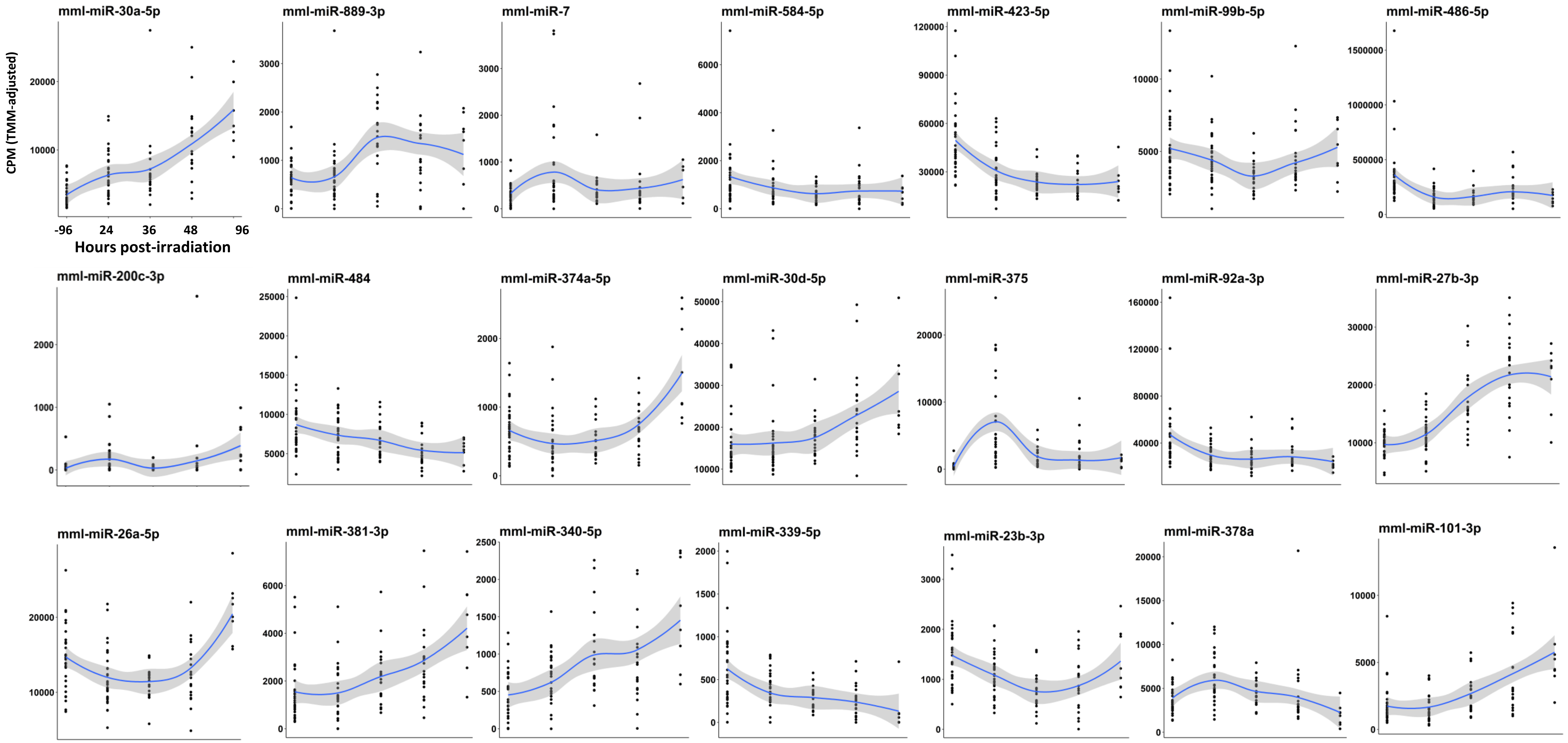

Supplemental Figure 2. Scatterplot and LOESS visualization of significantly modulated miRNAs (at any time point) that were detectable in at least 90% of the samples.

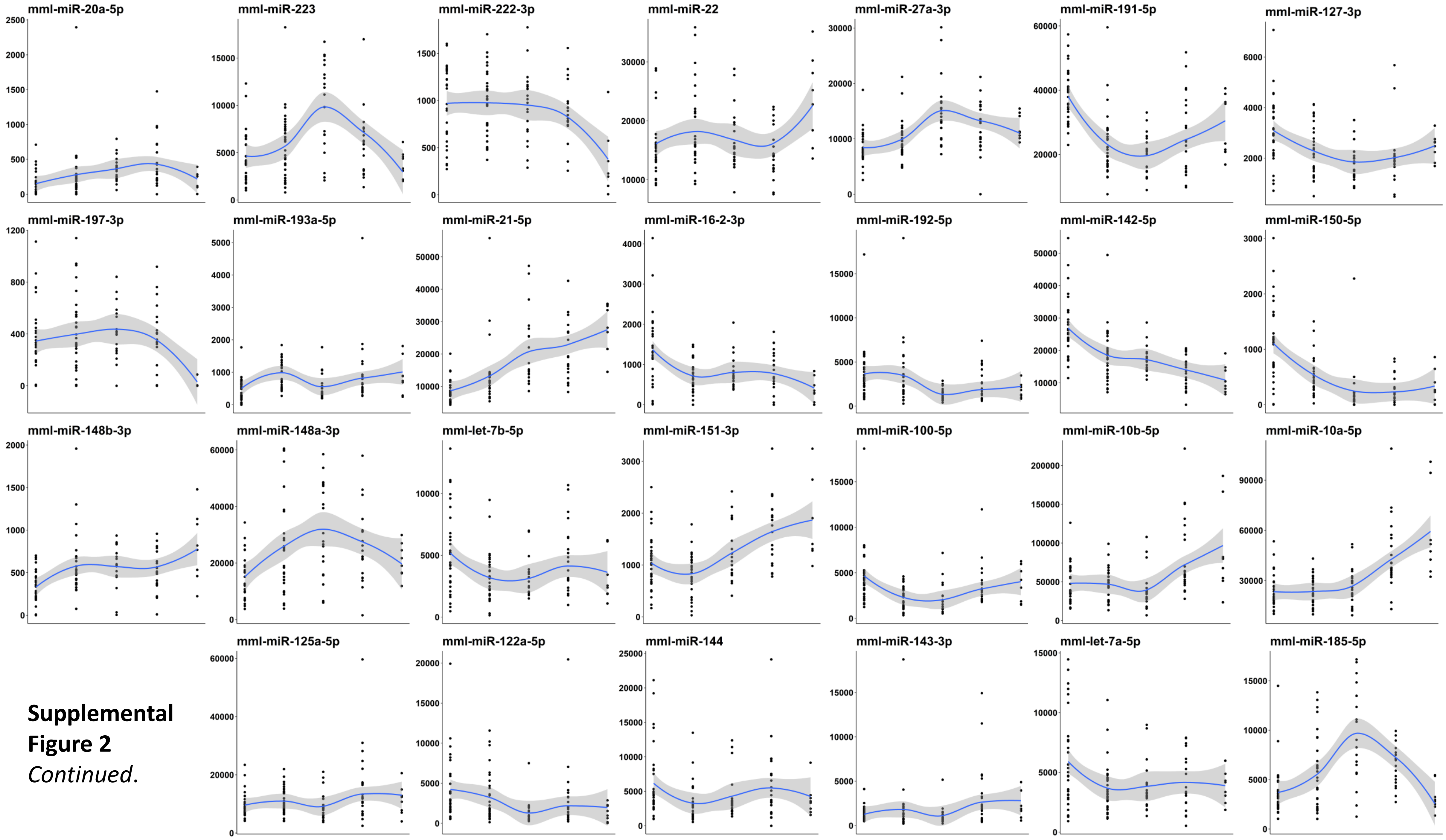

Supplemental Figure 2 *Continued*.

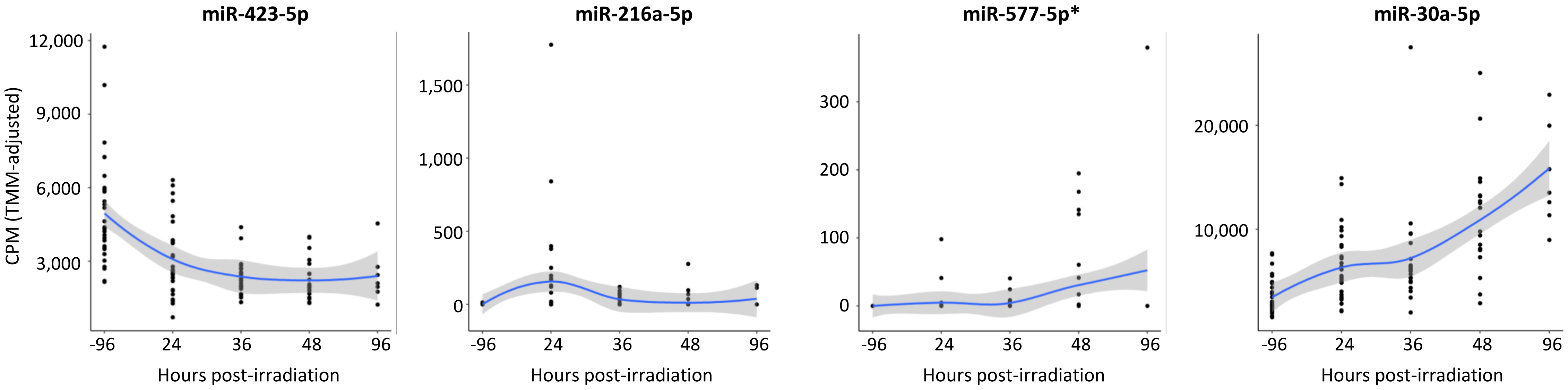

Supplemental Figure 3. Scatterplot and LOESS visualization of consistently (across all timepoints) significantly modulated miRNAs.
*****miRNA-577-5p is undetectable in all 30 pre-irradiation samples and displays an elevation following irradiation in a portion of samples. However, it did not meet our criteria of being detectable in at least 50% of the samples and was not included for analysis after Figure 1.

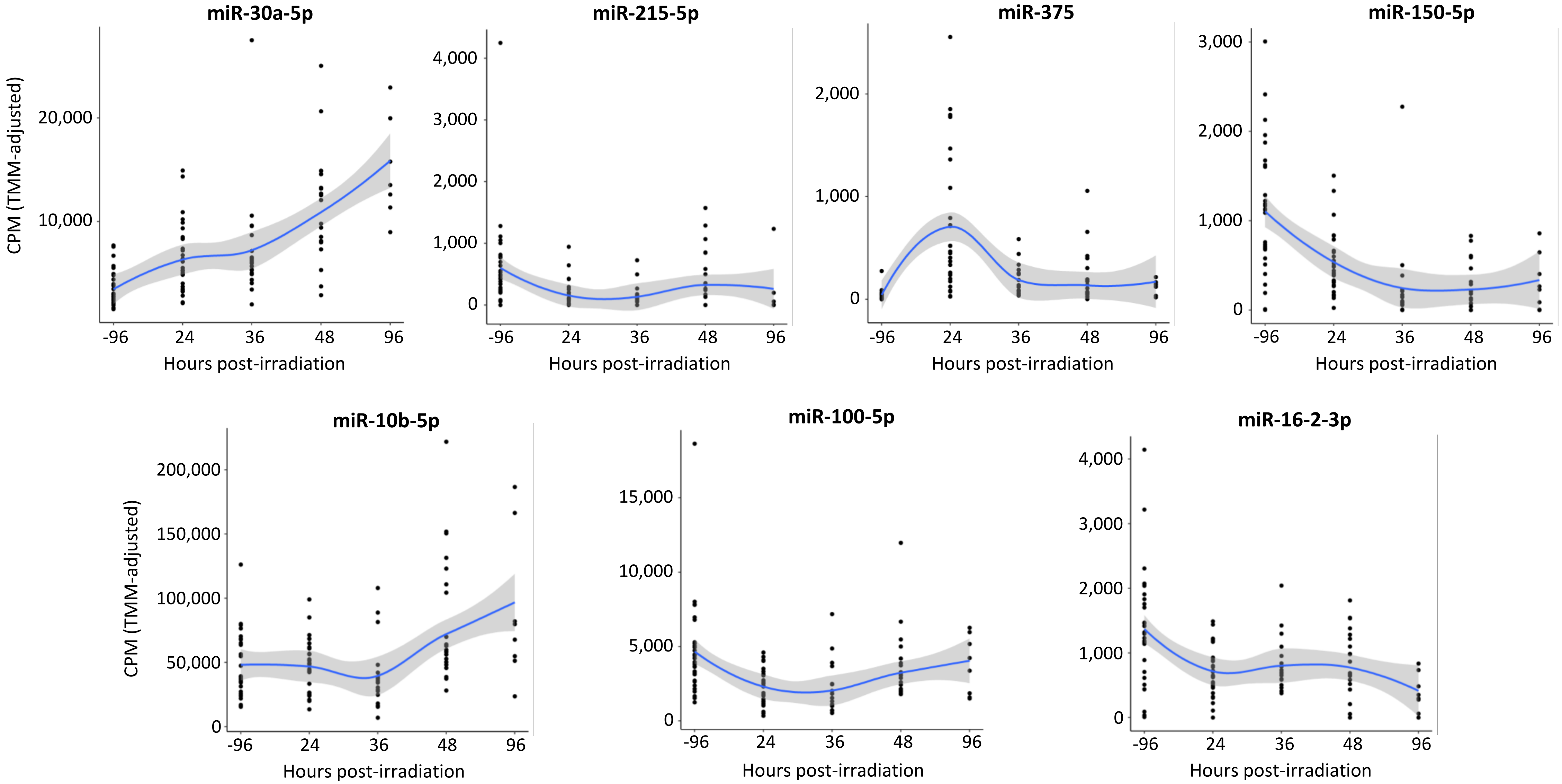

Supplemental Figure 4. Scatterplot and LOESS visualization of miRNAs previously found to be significantly modulated following irradiation.

Supplemental Table 1. Mean serum miRNA level across all conditions. Hours are in relation to the time from radiation exposure

miRNA	Untreated-96 h	Untreated 24 h	Untreated 36 h	Ex-RAD I 36 h	Untreated 48 h	Ex-Rad 48 h	Untreated 96 h	Ex-Rad I 96 h	Ex-Rad II 96 h
let-7a-3p	85	74	81	84	74	170	64	88	62
let-7a-5p	5913	3683	3832	2854	4356	2371	1079	4063	3113
let-7b-3p	95	98	79	73	124	94	62	102	30
let-7b-5p	5187	3194	3131	2658	4747	2992	1546	4495	3454
let-7c-5p	389	291	293	209	495	426	340	363	449
let-7d	1291	977	821	801	947	415	622	758	682
let-7e-3p	2	1	12	4	10	9	0	0	0
let-7e-5p	269	233	146	84	210	107	181	240	143
let-7f-3p	8	13	12	13	6	21	0	16	0
let-7f-5p	4692	3114	2986	2654	3306	1997	760	2798	2776
let-7g-5p	2605	3153	3009	3332	3407	2818	985	2591	2472
let-7i-5p	1905	2345	2335	2226	2554	2260	924	2469	2636
let-7i-3p	11	31	36	26	11	34	32	13	49
miR-1-3p	123	318	213	185	175	168	986	179	305
miR-100-5p	4665	2307	2049	1558	3675	3915	1665	2998	6476
miR-101-3p	1744	1671	2685	4095	4241	7069	2129	7358	5601
miR-103-3p	1581	2344	1684	1501	1619	1442	627	1816	1615
miR-106a-5p	1	17	3	12	1	15	0	0	1
miR-106b-5p	174	268	330	437	364	512	234	688	590
miR-106b-3p	213	184	161	360	224	228	120	214	127
miR-107-3p	1040	1333	1035	987	1014	1049	582	1225	1128
miR-10a-3p	70	135	142	127	149	287	328	175	247
miR-10a-5p	23427	23545	26734	22287	45648	46027	20146	53107	64018
miR-10b-5p	48096	46817	39498	38921	83270	101454	43738	103372	111478
miR-10b-3p	253	316	245	222	412	752	339	827	531
miR-1180-3p	130	77	111	161	167	320	134	225	93
miR-1185-3p	293	239	300	361	192	236	223	333	223
miR-1224-5p	9	51	15	6	11	26	0	25	36
miR-1225-5p	3	24	0	0	17	0	0	0	0
miR-1226	1	6	4	8	4	0	0	18	0
miR-122a-5p	4224	3230	1279	1200	3135	3746	886	2388	6170
miR-1230	22	0	6	3	8	5	15	0	0
miR-1234	20	12	4	4	2	0	0	0	0
miR-1247-5p	6	23	1	6	2	24	54	0	14
miR-1249	16	22	6	10	2	22	0	9	41
miR-124a-3p	2	36	11	2	2	0	0	0	0
miR-125a-5p	9597	10969	9195	8421	15918	13975	3895	16992	17152
miR-125a-3p	62	104	46	47	37	9	63	64	30
miR-125b-1-3p	13	6	8	2	9	33	60	0	25
miR-125b-5p	2641	2088	1712	1165	2204	2158	1050	3128	3741
miR-125b-2-3p	140	151	117	121	115	118	27	183	349
miR-126	2880	2663	3214	5202	3065	3833	1052	5050	3247
miR-1260b	169	146	158	152	105	43	192	107	135
miR-127-5p	25	11	34	45	0	34	5	19	10
miR-127-3p	3098	2285	1852	1595	2092	1855	394	2466	1985
miR-1271-5p	1168	1120	1524	1586	1344	1242	288	998	765
miR-1284	19	4	5	5	0	0	0	0	0
miR-128a-3p	856	898	1033	1293	924	1104	481	1031	946
miR-128b-3p	825	859	975	1162	808	1034	450	979	925
miR-129-5p	5	1	6	2	31	0	0	0	37
miR-1296-5p	119	86	71	66	104	104	0	57	84
miR-1304	119	54	103	61	94	162	111	146	84
miR-1306-5p	30	14	25	4	7	14	0	1	21
miR-130a-3p	164	254	201	185	86	199	89	78	67
miR-130b-5p	108	63	95	84	68	10	59	11	6
miR-130b-3p	73	123	98	101	171	41	76	115	28
miR-132-3p	30	64	69	27	54	172	63	152	17
miR-133a	47	110	28	59	30	78	198	81	105
miR-133b-3p	6	34	8	10	12	12	35	8	43
miR-133c-5p	4	21	17	28	29	17	0	66	53
miR-133c-3p	47	110	28	59	30	78	198	81	105
miR-134-3p	6	9	15	6	10	0	0	6	8
miR-134-5p	1706	2470	3103	2220	2161	1058	644	864	1076
miR-135a-1-3p	13	3	4	6	0	0	0	0	0
miR-136	16	7	13	53	8	19	0	11	50
miR-139-5p	129	227	159	196	133	303	166	379	192
miR-139-3p	30	45	27	22	29	10	0	78	22
miR-140-5p	25	16	51	86	36	120	107	78	0
miR-140-3p	10944	8541	11389	12099	9075	9318	1613	9028	8452
miR-141-3p	71	305	54	86	152	324	115	159	261
miR-142-5p	26914	18546	17187	21067	13606	16078	3567	15739	18400
miR-142-3p	55	106	39	39	54	47	197	48	68
miR-143-3p	1276	1817	1123	1665	3486	3237	1099	3914	7327
miR-144	6165	3278	4298	4565	6111	16631	1540	17597	13654
miR-145-5p	103	153	89	69	173	63	213	74	192

miR-145-3p	645	420	492	357	778	783	588	1761	2948
miR-146a-5p	7771	8002	5786	4282	6838	5918	5299	8813	8587
miR-146b-5p	650	557	286	231	644	508	491	377	381
miR-146b-3p	29	9	7	20	10	29	0	9	0
miR-148a-3p	15129	25966	31985	42679	25799	28016	4917	23362	24971
miR-148a-5p	1172	998	1681	1514	1264	1085	1068	1488	1375
miR-148b-5p	13	1	3	8	9	18	0	0	0
miR-148b-3p	332	578	572	737	520	909	315	640	501
miR-149-3p	11	7	3	1	33	1	39	0	0
miR-149-5p	25	23	10	11	55	13	0	60	64
miR-150-5p	1105	538	247	204	268	175	243	407	333
miR-150-3p	514	377	186	111	159	154	187	91	37
miR-151-5p	1703	1352	1095	1126	1097	1159	537	1799	1730
miR-151-3p	1038	831	1232	1963	1690	2281	591	2188	1609
miR-152-3p	532	950	582	668	618	882	275	867	908
miR-154-5p	4	1	9	4	0	3	0	0	0
miR-155	285	293	271	262	325	245	216	406	328
miR-15a-5p	154	169	116	150	86	132	98	142	243
miR-15a-3p	1	10	9	3	18	12	0	0	25
miR-15b-3p	302	186	245	272	265	289	144	283	204
miR-15b-5p	500	274	329	362	354	535	199	412	282
miR-16-5p	2461	1791	1669	2662	1906	2862	639	3291	2719
miR-16-2-3p	1370	717	803	918	826	987	228	570	1142
miR-17-5p	110	225	203	353	280	391	65	115	183
miR-17-3p	61	39	11	31	10	80	0	23	19
miR-181a-5p	2694	4633	5013	5204	4621	3741	1053	4772	2904
miR-181a-3p	30	70	27	54	83	37	44	236	30
miR-181b-5p	446	652	787	690	787	461	532	469	630
miR-181c-5p	37	47	20	85	52	35	68	34	25
miR-181c-3p	29	41	50	111	40	111	74	74	18
miR-181d	44	47	46	65	72	79	125	41	39
miR-182	41	105	21	35	44	34	101	75	98
miR-183-5p	12	55	14	23	33	35	108	33	48
miR-184	18	17	3	6	4	0	93	10	0
miR-185-5p	3708	5553	9687	9477	6216	5070	1084	4129	3855
miR-185-3p	34	33	19	31	51	6	44	0	0
miR-186-5p	2499	1862	2404	3460	3254	5793	521	3267	4226
miR-186-3p	27	6	1	20	8	0	0	0	0
miR-187-5p	314	206	202	155	292	101	237	94	99
miR-187-3p	326	300	492	512	317	379	353	366	452
miR-188-5p	13	35	16	15	3	12	0	33	0
miR-18a-3p	27	15	13	14	2	7	123	0	11
miR-18a-5p	7	33	7	10	6	15	20	37	0
miR-190b	1	3	6	4	3	2	0	0	6
miR-191-5p	38068	23107	19610	17823	26465	24811	8620	27404	29743
miR-191-3p	94	68	113	101	40	19	54	50	73
miR-192-5p	3656	3473	1342	1152	2552	2287	694	2556	2879
miR-193a-5p	502	985	555	509	1003	1070	402	660	681
miR-193a-3p	0	18	10	6	1	0	0	31	0
miR-193b-3p	30	41	21	6	27	47	20	36	23
miR-193b-5p	136	72	61	62	133	32	99	13	95
miR-194-5p	142	312	59	52	123	74	236	45	103
miR-195-5p	9	22	23	49	33	18	0	80	11
miR-195-3p	0	7	2	6	24	3	0	2	38
miR-196a-5p	1	16	4	2	11	4	0	14	63
miR-196b-5p	7	23	14	14	75	10	53	0	0
miR-197-3p	345	399	437	413	379	338	19	349	283
miR-197-5p	14	4	3	5	10	0	0	0	0
miR-199a-5p	356	287	196	235	412	403	143	268	369
miR-199a	356	287	196	235	412	403	143	268	369
miR-199a-3p	3305	2815	3221	3939	3219	4028	941	4242	4640
miR-199a2	356	287	196	235	410	403	143	268	369
miR-19a-3p	126	115	147	210	83	189	135	105	120
miR-19b	557	428	374	511	254	640	196	411	569
miR-19b2	557	428	374	510	254	640	196	411	569
miR-200a-3p	50	509	86	135	323	299	301	327	719
miR-200c-3p	29	376	30	55	179	62	305	39	57
miR-203	203	267	194	123	167	283	560	148	193
miR-204-3p	10	1	2	6	0	24	0	0	0
miR-204-5p	23	10	5	13	12	1	0	14	28
miR-205	40	55	52	36	90	122	2	78	54
miR-206	123	178	68	99	118	232	90	84	119
miR-208b-3p	3	28	5	10	0	0	0	0	0
miR-20a-5p	151	279	366	615	491	816	121	340	624
miR-21-3p	446	367	595	622	480	1119	421	1149	596
miR-21-5p	8660	13228	20734	26624	21139	31455	6031	32250	28112
miR-210-3p	162	216	273	342	208	331	231	151	120

miR-212-3p	2	11	2	4	17	12	58	2	19
miR-214-3p	395	153	111	70	169	149	79	165	131
miR-215-5p	603	155	134	137	437	166	265	262	225
miR-216a-3p	0	17	2	6	4	3	0	0	0
miR-216a-5p	1	157	35	24	24	23	46	13	67
miR-216b	0	61	14	17	5	0	0	0	25
miR-217	0	209	56	47	39	19	1	15	84
miR-218-5p	3	3	6	0	2	0	33	0	0
miR-22	16113	18185	16775	13956	14741	18978	6070	20793	20830
miR-221-5p	71	79	114	127	131	242	179	335	281
miR-221-3p	3902	4412	5091	4953	3232	3609	689	2839	3445
miR-222-3p	971	976	952	1024	875	626	249	788	401
miR-223	4650	5705	9841	14339	5886	6916	1161	4594	4514
miR-224-5p	16	45	18	46	114	45	22	12	0
miR-23a-5p	51	18	23	25	43	12	94	26	44
miR-23a-3p	6346	6079	7428	7833	7956	10426	2545	10361	10132
miR-23b-5p	66	96	45	47	104	111	121	126	32
miR-23b-3p	1478	1085	756	720	891	1323	544	1314	1047
miR-24-3p	37699	33433	29496	23953	36118	41277	10493	55952	46679
miR-24-5p	10	4	6	6	0	4	0	13	52
miR-25	4215	3278	4951	7278	5616	7759	675	6654	5949
miR-26a-1-3p	1	0	9	13	3	0	0	0	0
miR-26a-5p	14718	11998	11440	9426	12556	12625	3073	16461	16786
miR-26b-3p	144	87	121	138	133	152	0	78	79
miR-26b-5p	1232	1695	1241	1300	1217	1133	525	1076	1004
miR-27a-5p	27	34	17	19	11	20	0	33	35
miR-27a-3p	8407	9943	15082	15921	11738	18795	2001	17016	10561
miR-27b-5p	2	1	2	7	16	3	100	0	0
miR-27b-3p	9702	11471	17854	23282	22135	30534	4360	26824	20863
miR-28-5p	76	118	84	184	113	100	122	99	60
miR-28-3p	631	563	767	778	587	721	258	631	665
miR-296-3p	323	459	296	247	353	150	78	323	88
miR-296-5p	3	7	2	10	1	12	0	0	0
miR-299-5p	19	31	13	26	4	16	0	4	7
miR-299-3p	709	686	958	1217	682	825	454	1002	849
miR-29a-3p	2752	4159	2780	3123	2913	4423	1464	4025	3447
miR-29b-3p	204	181	137	94	81	100	56	211	167
miR-29b-2-5p	4	6	7	1	0	0	0	0	0
miR-29c-3p	494	1008	437	538	486	657	278	762	646
miR-30a-3p	74	132	166	244	255	455	131	170	233
miR-30a-5p	3469	6336	7231	8897	11252	21830	4021	18809	16784
miR-30b-5p	306	257	171	224	221	163	185	83	152
miR-30b-3p	17	3	4	5	34	0	0	12	0
miR-30c-1-3p	6	2	15	4	4	8	77	0	0
miR-30c-5p	1863	1622	1286	1188	1362	1293	300	1028	1586
miR-30c-2-3p	15	3	8	5	1	4	46	14	7
miR-30d-3p	9	9	37	37	17	48	79	28	0
miR-30d-5p	15976	16205	17529	21160	23736	35570	8610	32699	23989
miR-30e-5p	6946	6003	6533	9172	8311	18144	3452	13792	9875
miR-30e-3p	104	65	114	168	139	148	128	168	262
miR-31-5p	10	16	2	1	0	1	54	0	0
miR-3122	8	10	14	21	33	7	37	0	34
miR-3173	6	4	5	9	27	1	0	0	0
miR-32-5p	167	103	154	129	101	199	204	145	282
miR-32-3p	8	3	0	4	0	8	11	0	0
miR-320a	29781	28855	22262	13185	22616	12410	5637	16642	17257
miR-320b	2366	2537	1754	1108	2668	1166	1017	2238	1567
miR-320c	57	58	42	28	23	11	62	1	38
miR-323a-3p	445	365	475	367	238	139	159	165	261
miR-323a-5p	8	12	4	13	0	10	0	0	0
miR-323b-3p	1505	1113	1330	928	669	451	463	545	625
miR-324-5p	2	3	6	11	21	19	0	0	0
miR-324-3p	15	4	9	15	6	5	23	10	0
miR-328	39	42	21	22	42	15	64	8	57
miR-329-3p	114	109	147	185	95	86	171	49	135
miR-330-3p	179	231	176	180	141	86	53	44	127
miR-331-3p	141	116	117	134	98	143	107	44	141
miR-331-5p	1110	696	1049	876	1159	2631	722	1779	1532
miR-335-5p	294	248	218	154	179	221	250	199	247
miR-335-3p	18	11	5	3	14	12	0	0	15
miR-337-5p	145	61	97	93	90	72	166	164	49
miR-337-3p	57	38	64	76	27	58	84	78	56
miR-338-3p	12	11	8	13	4	33	0	37	0
miR-338-5p	167	213	171	135	239	193	166	239	75
miR-339-5p	625	345	290	321	250	543	147	171	339
miR-339-3p	789	582	636	573	598	620	407	778	905
miR-340-5p	453	622	989	1555	910	1600	616	1210	1049

miR-340-3p	44	39	21	32	30	0	41	0	0
miR-342-3p	629	1054	331	268	286	177	156	144	245
miR-342-5p	65	38	15	9	24	0	0	61	11
miR-34a-5p	7	42	6	16	19	0	69	85	12
miR-34c-5p	8	24	4	5	0	12	0	0	0
miR-361-3p	92	181	93	116	110	100	14	87	88
miR-361-5p	2382	1962	3147	2425	3154	3611	790	3249	2941
miR-362-3p	2	9	4	1	5	8	0	0	20
miR-362-5p	11	19	14	10	3	2	47	4	34
miR-363-3p	293	414	402	437	259	253	161	190	255
miR-365-1-5p	6	8	0	5	0	0	17	0	0
miR-365-3p	4	35	13	7	15	14	0	15	45
miR-365-2-5p	1	9	4	1	3	13	0	3	18
miR-369-3p	695	746	1189	1199	724	382	589	576	743
miR-369-5p	6	9	9	21	10	0	0	40	11
miR-370-3p	1534	1274	1388	1470	1616	1348	780	1288	878
miR-371-5p	7	6	7	21	17	2	0	0	0
miR-374a-3p	5	22	8	20	1	0	0	0	29
miR-374a-5p	661	470	512	653	677	1154	630	1333	1077
miR-374b-3p	2	16	2	11	3	0	0	9	8
miR-374b-5p	66	67	69	116	108	64	114	110	32
miR-375	424	7064	1876	1982	2237	1697	625	1630	3916
miR-376a-3p	15	14	38	34	8	34	72	0	0
miR-376c-3p	191	178	249	312	167	174	107	183	100
miR-377-3p	302	325	407	408	174	302	332	314	219
miR-377-5p	54	36	61	54	15	40	0	17	9
miR-378a	3906	5923	4651	3955	4341	3157	930	2863	2465
miR-378d	467	854	700	668	932	456	135	508	317
miR-379-5p	102	96	163	226	154	104	280	71	168
miR-379-3p	24	22	21	43	16	14	76	0	0
miR-380-3p	471	348	562	310	279	133	235	166	155
miR-381-3p	1552	1501	2187	3012	2705	4712	1536	3682	2957
miR-382-5p	741	748	1199	1257	711	420	459	499	692
miR-382-3p	344	276	334	404	195	127	238	114	155
miR-409-3p	5051	4627	7580	9215	5414	4239	1605	4597	5211
miR-409-5p	565	396	445	425	405	420	414	446	418
miR-410-3p	589	623	1322	1245	788	747	618	1042	947
miR-411-3p	73	81	83	113	80	69	145	91	120
miR-411-5p	69	64	84	134	103	134	76	26	90
miR-412-5p	18	9	18	10	15	0	61	11	0
miR-421	115	74	98	91	43	31	24	7	92
miR-423-3p	9024	11624	9823	9590	7677	7409	1566	6743	7116
miR-423-5p	49551	30864	23750	14426	22759	15188	6661	20400	27571
miR-424-3p	45	35	31	47	39	38	34	73	125
miR-424-5p	73	119	136	161	131	181	121	130	269
miR-425	1116	1016	1310	1416	1101	935	328	1234	882
miR-429-3p	12	54	11	23	12	1	0	44	12
miR-431	62	64	101	104	63	34	45	64	58
miR-432-5p	565	422	884	1291	869	807	157	492	469
miR-433-3p	247	220	395	506	317	318	172	169	216
miR-4446-3p	209	244	317	379	360	273	194	258	176
miR-450a-5p	16	22	13	11	15	16	49	83	63
miR-450b-5p	110	102	128	120	210	226	174	101	66
miR-451	9058	7441	5952	7801	6656	11308	3547	9951	12306
miR-452-5p	70	108	81	76	161	251	119	62	118
miR-452-3p	1	17	5	15	9	17	0	0	0
miR-455-3p	53	10	9	19	3	6	39	17	9
miR-455-5p	44	29	18	19	53	44	83	10	92
miR-4667	14	17	3	4	6	0	48	0	0
miR-4677-5p	0	25	3	0	3	45	0	0	0
miR-4743	5	7	3	0	5	0	0	0	0
miR-4766-3p	2	1	2	6	3	6	0	0	0
miR-484	8691	7352	6692	4688	5056	4106	1171	4883	3646
miR-485-5p	302	210	255	248	363	141	142	215	223
miR-485-3p	270	186	193	211	204	126	160	157	166
miR-486-5p	362126	160352	164007	115392	235276	262605	43400	211951	336737
miR-486-3p	758	377	253	261	311	204	90	198	156
miR-487b-3p	154	136	163	163	94	119	113	79	32
miR-491-5p	5	14	5	2	0	0	0	21	0
miR-493-5p	227	130	186	297	228	251	305	69	169
miR-493-3p	84	36	99	143	116	122	46	116	78
miR-494-3p	260	265	392	554	208	320	113	236	270
miR-495-3p	202	226	270	348	200	80	176	190	191
miR-496	75	66	130	125	33	48	75	67	42
miR-497-5p	16	80	39	19	76	10	0	45	40
miR-499-5p	67	164	64	125	81	189	150	197	95
miR-500a-3p	1395	1443	1192	854	1286	1625	753	1761	1091

miR-500b-3p	110	128	61	68	109	56	47	124	92
miR-501-3p	110	129	62	68	110	56	47	124	92
miR-502-3p	466	588	418	438	363	487	324	626	344
miR-503-5p	78	99	70	70	65	49	132	75	119
miR-505-5p	8	0	8	2	0	0	0	42	12
miR-505-3p	42	52	44	57	47	78	58	52	38
miR-506-3p	1	58	10	15	40	0	46	0	0
miR-511-5p	0	14	5	6	9	5	0	0	0
miR-511-3p	9	5	4	1	3	10	0	0	0
miR-532-3p	45	37	30	29	24	0	69	0	26
miR-532-5p	451	469	277	472	336	465	321	324	117
miR-541-3p	183	157	222	163	144	68	161	95	66
miR-541-5p	0	1	4	14	1	7	0	0	5
miR-542-3p	5	31	15	37	28	31	0	48	55
miR-543-3p	1068	794	1313	1486	623	583	318	702	605
miR-550-5p	27	15	30	21	12	0	0	1	20
miR-551a	13	29	16	2	6	22	0	0	19
miR-574	379	395	228	279	237	237	40	235	220
miR-576-3p	75	109	73	67	58	119	54	104	51
miR-577-5p	0	5	4	7	38	34	83	0	37
miR-582-3p	3	11	20	22	7	45	30	41	35
miR-584-5p	1339	870	626	461	847	832	289	1062	919
miR-590-3p	22	24	44	96	46	88	0	79	10
miR-598-3p	38	74	110	135	65	85	204	144	21
miR-599-5p	2	11	4	2	16	0	0	0	0
miR-601	14	30	5	0	0	9	0	0	0
miR-605	203	166	145	134	109	42	165	108	62
miR-6127	2	7	6	9	0	1	0	0	10
miR-615-3p	85	21	20	24	19	23	42	0	92
miR-627-5p	367	532	669	404	356	368	280	292	404
miR-628-3p	251	261	272	264	309	347	113	286	191
miR-628-5p	2	4	4	3	11	2	0	0	0
miR-638	6	26	5	2	104	0	0	7	0
miR-652	286	223	208	240	137	241	161	320	191
miR-6529-5p	5574	4234	5101	3252	3193	1516	928	1957	2581
miR-6529-3p	49	27	29	48	9	33	57	12	28
miR-654-5p	81	82	148	184	81	164	48	82	115
miR-654-3p	9858	7257	12247	9877	6869	5682	3883	7209	4479
miR-656-3p	823	641	1019	941	476	386	267	360	448
miR-656-5p	5	9	15	8	16	18	0	0	6
miR-660-5p	125	127	187	196	166	248	189	216	195
miR-663	10	34	6	3	2	0	8	1	0
miR-664	6	0	4	7	13	59	98	81	20
miR-665	371	291	364	278	167	166	145	244	73
miR-668	54	26	39	31	30	14	21	43	11
miR-671-3p	73	87	70	84	63	94	145	35	61
miR-671-5p	7	17	23	14	1	0	0	0	0
miR-675-3p	3	18	1	3	15	0	0	0	0
miR-675-5p	2	19	2	1	11	6	0	0	0
miR-676-3p	40	44	36	62	64	178	120	57	47
miR-6827-3p	17	7	12	10	8	8	37	0	13
miR-7	326	784	404	393	500	511	320	429	506
miR-7170-3p	5	8	6	1	8	0	0	24	0
miR-7174-3p	16	246	61	17	31	65	0	191	24
miR-7174-5p	9	14	12	7	0	7	0	21	16
miR-7176-5p	4	33	0	0	5	3	0	5	0
miR-7177-3p	4	12	7	13	0	103	0	28	0
miR-7180-5p	38	17	50	34	53	27	77	0	4
miR-7180-3p	21	2	13	18	35	0	0	0	0
miR-7184-3p	9	19	6	9	17	10	0	0	0
miR-7186-3p	5	0	4	6	3	18	0	0	0
miR-7186-5p	116	32	90	72	88	114	63	86	27
miR-7204-3p	23	7	5	11	10	0	45	63	0
miR-7205-3p	40	24	56	29	23	0	18	23	0
miR-7205-5p	3	12	8	2	0	13	0	0	0
miR-758-3p	66	61	22	39	7	30	134	47	33
miR-760	489	433	518	425	372	241	268	202	225
miR-761	0	57	4	0	0	0	0	0	0
miR-769-5p	32	35	29	20	13	29	0	24	0
miR-802	0	44	18	17	3	9	0	20	0
miR-873-5p	1	37	7	5	7	7	0	9	0
miR-873-3p	6	15	2	2	5	0	33	6	0
miR-874-3p	99	143	92	73	107	78	20	132	72
miR-877-5p	467	232	158	131	204	96	265	209	179
miR-885-5p	78	54	31	25	15	22	0	12	0
miR-889-3p	641	661	1475	1750	1149	1142	610	1266	810
miR-9-3-3p	45	19	41	99	15	15	98	22	0

miR-9-3p	45	19	41	99	15	15	98	22	0
miR-9-5p	26	39	10	13	11	52	39	11	0
miR-92a-3p	47104	29723	26266	24044	30977	26090	3751	24927	32521
miR-92b-3p	661	796	463	315	684	354	171	555	405
miR-93-5p	508	498	716	1001	807	975	497	769	892
miR-93-3p	89	185	158	154	73	77	0	37	83
miR-933	5	3	3	2	0	13	0	0	0
miR-939	4	8	5	2	2	3	0	0	0
miR-940	3	2	4	17	7	0	0	0	76
miR-942-3p	45	7	7	19	17	13	0	0	0
miR-942-5p	269	135	128	216	91	131	150	109	190
miR-95-3p	38	75	25	74	50	45	79	19	127
miR-96	48	149	15	22	18	38	56	15	90
miR-98	128	82	85	111	119	68	67	146	114
miR-99a-5p	26665	20819	17556	14796	33018	36489	8625	39202	56421
miR-99b-3p	5	30	15	17	18	24	0	31	50
miR-99b-5p	5205	4400	3298	3052	4666	4450	1658	4614	5838

Supplemental Table 2. Significantly modulated miRNAs per timepoint.

Note: Siginifcantly modulated miRNAs are highlighted in red. Only miRNAs with a log2FC of (>.58) and a false discovery rate-adjujsted p-value of (<0.05) were considered significant.

24 Houst Post-Irradiation				36 Houst Post-Irradiation				48 Houst Post-Irradiation				96 Houst Post-Irradiation			
miRNA	log2FC	PValue	FDR	miRNA	log2FC	PValue	FDR	miRNA	log2FC	PValue	FDR	miRNA	log2FC	PValue	FDR
miR-375	4.06	1.52E-20	5.54E-18	miR-21-5p	1.25	0.000000	0.000001	miR-30a-5p	1.69	2.39E-14	8.71E-12	miR-30a-5p	2.28	1.13E-16	4.10E-14
miR-217	9.83	6.63E-12	1.21E-09	miR-217	7.93	0.000000	0.000001	miR-27b-3p	1.19	1.20E-13	2.19E-11	miR-21-5p	1.82	4.00E-12	7.27E-10
miR-486-5p	-1.17	1.87E-09	2.27E-07	miR-185-5p	1.40	0.000000	0.000001	miR-21-5p	1.31	3.02E-10	3.66E-08	miR-27b-3p	1.22	5.52E-09	6.70E-07
miR-216a-5p	7.57	2.56E-07	2.33E-05	miR-423-5p	-1.04	0.000000	0.000003	miR-423-5p	-1.09	6.20E-09	5.65E-07	miR-10a-5p	1.38	1.46E-08	1.12E-06
miR-200a-3p	3.26	3.97E-06	0.000258593	miR-27b-3p	0.88	0.000000	0.000004	miR-217	7.51	1.39E-08	1.01E-06	miR-26b-3p	-9.80	1.54E-08	1.12E-06
miR-100-5p	-1.07	4.26E-06	0.000258593	miR-191-5p	-0.94	0.000000	0.000014	miR-375	2.43	1.89E-08	1.15E-06	miR-101-3p	1.83	7.87E-08	4.69E-06
miR-191-5p	-0.72	6.01E-06	0.00028191	miR-375	2.16	0.000000	0.000025	miR-142-5p	-0.97	1.60E-07	8.31E-06	miR-93-3p	-8.22	9.03E-08	4.69E-06
miR-802	8.09	6.20E-06	0.00028191	miR-30a-5p	1.09	0.000001	0.000035	miR-10a-5p	0.94	7.70E-07	3.50E-05	miR-197-3p	-4.72	1.52E-06	6.90E-05
miR-216b	7.80	1.32E-05	0.000514998	miR-486-5p	-1.10	0.000001	0.000035	miR-101-3p	1.30	1.37E-06	5.55E-05	miR-1296-5p	-8.57	1.17E-05	0.000475162
miR-761	8.48	1.41E-05	0.000514998	miR-192-5p	-1.38	0.000003	0.000127	miR-143-3p	1.43	1.04E-05	0.000377817	miR-577-5p	8.37	1.53E-05	0.000555904
miR-423-5p	-0.70	1.86E-05	0.000614925	miR-100-5p	-1.22	0.000006	0.000194	miR-577-5p	7.85	1.29E-05	0.000425677	miR-142-5p	-1.14	2.07E-05	0.000683567
miR-30a-5p	0.85	2.72E-05	0.000826227	miR-122a-5p	-1.75	0.000012	0.000337	miR-150-5p	-2.03	3.84E-05	0.00116431	miR-340-5p	1.88	7.06E-05	0.002143013
miR-92a-3p	-0.67	4.06E-05	0.001135441	miR-92a-3p	-0.81	0.000012	0.000337	miR-127-5p	-7.31	4.93E-05	0.001379183	miR-200a-3p	3.42	8.46E-05	0.002370142
miR-181a-5p	0.75	8.55E-05	0.002221906	miR-181a-5p	0.91	0.000013	0.000343	miR-484	-0.75	0.000134249	0.003490473	miR-381-3p	1.56	0.000100438	0.002611393
miR-200c-3p	3.57	0.000125793	0.003052581	miR-150-5p	-2.15	0.000015	0.000357	miR-200a-3p	2.76	0.000157626	0.003825066	miR-30d-5p	0.92	0.000131511	0.00319134
miR-7174-3p	3.93	0.000353021	0.008031227	miR-223	1.07	0.000027	0.000622	miR-20a-5p	1.75	0.000183449	0.004173455	miR-885-5p	-8.84	0.000152245	0.003463569
miR-577-5p	4.92	0.000573839	0.012286911	miR-148a-3p	1.06	0.000035	0.000748	miR-181a-5p	0.77	0.000213645	0.00457451	miR-590-3p	-7.13	0.000242744	0.004844928
miR-193a-5p	0.97	0.000750406	0.014445838	miR-802	6.84	0.000046	0.000936	miR-7174-5p	-5.85	0.000236099	0.004774449	miR-342-5p	-8.61	0.000249358	0.004844928
miR-142-5p	-0.54	0.000754041	0.014445838	miR-216a-5p	5.27	0.000098	0.001809	miR-216a-5p	4.76	0.000316685	0.006067024	miR-377-5p	-8.40	0.000252895	0.004844928
miR-1230	-7.03	0.00104777	0.018782724	miR-27a-3p	0.83	0.000099	0.001809	miR-10b-5p	0.77	0.00050772	0.009240504	miR-574	-3.80	0.000292199	0.005318015
miR-148a-3p	0.76	0.001105124	0.018782724	miR-216b	5.65	0.000533	0.009244	miR-1284	-6.94	0.000664847	0.011524016	miR-423-5p	-0.96	0.000318918	0.005527911
miR-506-3p	6.14	0.001148348	0.018782724	miR-23b-3p	-0.94	0.000561	0.009283	miR-193a-5p	1.05	0.000853301	0.014118254	miR-92a-3p	-0.95	0.000371	0.006024057
let-7a-5p	-0.72	0.00118682	0.018782724	miR-142-5p	-0.61	0.000796	0.012602	miR-491-5p	-4.98	0.001027777	0.015594997	miR-216a-5p	5.29	0.000380641	0.006024057
miR-144	-0.91	0.001516848	0.022092831	miR-577-5p	4.77	0.000885	0.013429	miR-125a-5p	0.70	0.001028242	0.015594997	miR-222-3p	-1.34	0.000486413	0.007377271
miR-215-5p	-1.91	0.001517365	0.022092831	miR-99b-5p	-0.65	0.001447	0.021064	miR-323a-5p	-5.75	0.001267109	0.018449112	miR-486-5p	-1.10	0.000620545	0.009035132
miR-7	1.22	0.001786264	0.025007699	miR-127-3p	-0.73	0.001614	0.022596	miR-30a-3p	1.75	0.00165148	0.022973318	miR-486-3p	-3.27	0.000647663	0.009067287
miR-378a	0.62	0.002495656	0.033645136	miR-584-5p	-1.08	0.001868	0.025177	miR-185-5p	0.76	0.001704065	0.022973318	miR-10b-5p	0.99	0.00071585	0.009322817
let-7b-5p	-0.73	0.002667382	0.034675962	miR-761	4.62	0.001937	0.025177	miR-802	4.17	0.001846193	0.024000509	miR-1-3p	2.77	0.000729005	0.009322817
miR-21-5p	0.56	0.002944111	0.03695367	miR-340-5p	1.12	0.002927	0.036645	miR-92a-3p	-0.57	0.002159911	0.027110605	miR-374a-5p	1.33	0.000742752	0.009322817
miR-16-2-3p	-0.95	0.003306411	0.04011779	miR-889-3p	1.20	0.003020	0.036645	miR-30d-5p	0.56	0.002293208	0.027764302	miR-200c-3p	3.76	0.000873975	0.010604228
miR-29c-3p	1.06	0.004697276	0.055155112	miR-215-5p	-2.06	0.003379	0.039673	miR-191-5p	-0.55	0.002369798	0.027764302	miR-10a-3p	2.66	0.000960185	0.011231799
let-7f-5p	-0.64	0.005268262	0.059926479	miR-20a-5p	1.32	0.004761	0.054160	miR-506-3p	5.74	0.002440818	0.027764302	miR-7174-3p	-6.62	0.000987411	0.011231799
miR-29a-3p	0.57	0.006410238	0.07070687	miR-877-5p	-1.57	0.005328	0.058772	miR-29b-2-5p	-4.87	0.002575503	0.028408581	miR-148b-3p	1.44	0.001099988	0.012133201
miR-873-5p	5.11	0.007215937	0.077252974	miR-410-3p	1.16	0.007285	0.077988	miR-148a-3p	0.76	0.002826231	0.030257298	miR-497-5p	-6.76	0.001497681	0.016033998
miR-141-3p	2.21	0.007609707	0.079140952	miR-486-3p	-1.55	0.007657	0.079632	miR-7205-5p	-4.07	0.004029887	0.041910823	miR-296-3p	-2.67	0.001794685	0.018511225
miR-148b-3p	0.82	0.00879227	0.088899623	miR-146b-5p	-1.12	0.009017	0.086575	miR-200c-3p	2.70	0.004375076	0.044236879	miR-16-2-3p	-1.73	0.001830781	0.018511225
miR-185-5p	0.57	0.009818089	0.096588766	miR-99a-5p	-0.59	0.009102	0.086575	miR-216b	4.27	0.004513989	0.044407896	miR-17-3p	-8.59	0.001906146	0.018752352
miR-378d	0.87	0.012174628	0.116620119	miR-134-5p	0.87	0.009226	0.086575	miR-486-5p	-0.61	0.005406897	0.050960449	miR-339-5p	-2.29	0.002376244	0.022761916
miR-150-5p	-1.03	0.013122024	0.122472223	miR-151-5p	-0.63	0.009276	0.086575	miR-378d	1.06	0.005460048	0.050960449	miR-26a-5p	0.63	0.002610389	0.024363626
miR-7186-5p	-1.90	0.014495792	0.13191171	miR-214-3p	-1.80	0.010219	0.092990	miR-151-3p	0.68	0.006298117	0.055509701	miR-942-3p	-8.18	0.002981838	0.027134727
miR-152-3p	0.85	0.018892814	0.167731321	miR-125b-5p	-0.62	0.011737	0.104201	miR-638	4.18	0.006401713	0.055509701	miR-550-5p	-7.22	0.004016843	0.035661732
miR-214-3p	-1.40	0.019670505	0.170019195	let-7b-5p	-0.66	0.016159	0.137270	miR-23b-3p	-0.74	0.006404966	0.055509701	miR-1306-5p	-7.56	0.004221108	0.036562934
miR-103-3p	0.51	0.020174175	0.170019195	let-7f-5p	-0.63	0.016216	0.137270	miR-340-5p	1.01	0.007071209	0.05985861	miR-1224-5p	-5.70	0.004490885	0.038015868
miR-331-5p	-0.65	0.020551771	0.170019195	miR-150-3p	-1.49	0.017393	0.143891	miR-150-3p	-1.69	0.007391989	0.06115191	miR-22	0.64	0.004696614	0.038853807
miR-423-3p	0.36	0.021050418	0.170274491	miR-148b-3p	0.81	0.017991	0.145525	miR-151-5p	-0.65	0.007602918	0.061499162	miR-122a-5p	-1.61	0.005246579	0.042246106
miR-1-3p	1.41	0.022747569	0.177330081	miR-181b-5p	0.85	0.019447	0.153882	miR-339-5p	-1.28	0.008447404	0.066844676	miR-151-3p	0.92	0.005338794	0.042246106
miR-140-3p	-0.35	0.022897016	0.177330081	miR-101-3p	0.62	0.020896	0.159272	miR-601	-6.38	0.00865124	0.067001091	miR-139-3p	-6.27	0.005606724	0.04342229
miR-25	-0.38	0.023864735	0.180974238	let-7a-5p	-0.58	0.021003	0.159272	miR-6529-5p	-0.80	0.010172873	0.077144291	miR-10b-3p	1.46	0.007719365	0.058538518
miR-365-3p	3.17	0.025559803	0.189872819	miR-339-5p	-1.05	0.029637	0.220160	miR-381-3p	0.80	0.010443136	0.07757758	miR-125b-2-3p	-3.18	0.008682373	0.064497626
miR-1224-5p	2.52	0.026603284	0.193671909	miR-26a-5p	-0.34	0.032389	0.235778	miR-323b-3p	-1.13	0.011628093	0.084652519	miR-375	1.42	0.00961799	0.070018964
miR-26a-5p	-0.31	0.027621122	0.197138991	miR-506-3p	3.66	0.033325	0.235778	miR-19b	-1.11	0.012823677	0.091525854	miR-92b-3p	-1.38	0.010195528	0.072768081
miR-486-3p	-1.09	0.029212001	0.204484007	miR-30a-3p	1.18	0.034309	0.235778	miR-758-3p	-3.16	0.013641725	0.095492072	miR-506-3p	5.18	0.010844508	0.075462759
miR-127-3p	-0.44	0.031380581	0.215519462	miR-106b-5p	0.98	0.034500	0.235778	miR-196b-5p	3.55	0.017079638	0.117301666	miR-27a-5p	-7.33	0.010987709	0.075462759
miR-942-3p	-2.86	0.033881371	0.228385541	miR-30c-5p	-0.49	0.034978	0.235778	miR-181b-5p	0.86	0.018730835	0.1262597	miR-205	-3.89	0.011221914	0.07564401
miR-186-5p	-0.44	0.035686158	0.23617748	miR-24-3p	-0.34	0.045657	0.297216	miR-342-3p	-1.19	0.01951555	0.12915746	miR-299-5p	-6.92	0.011552051	0.076453574
miR-877-5p	-1.00	0.038749363	0.251870856	miR-16-2-3p	-0.73	0.045726	0.297216	miR-25	0.43	0.019896034	0.129324219	miR-551a	-6.26	0.012608701	0.081956553
miR-339-5p	-0.86	0.042433842	0.261667199	miR-409-3p	0.57	0.046935	0.299728	miR-127-3p	-0.53	0.020891776	0.133195677	miR-149-5p	-7.22	0.013616178	0.086952432
miR-542-3p	2.81	0.04281505	0.261667199	miR-221-3p	0.40	0.048137	0.302100	miR-106b-5p	1.07	0.021223487	0.133195677	miR-410-3p	1.37	0.014616036	0.090498143
miR-196a-5p	3.66	0.043071932	0.261667199					miR-27a-3p	0.49	0.021836649	0.134721024	miR-30a-3p	1.72	0.014806948	0.090498143
miR-584-5p	-0.61	0.043193785	0.261667199					miR-17-5p	1.43	0.025962114	0.157503494	miR-214-3p	-2.66	0.014917276	0.090498143
miR-15b-5p	-0.85	0.043850822	0.261667199					miR-486-3p	-1.27	0.026526287	0.15828801	miR-1249	-6.60	0.015689785	0.091957894
miR-7180-3p	-3.54	0.047491029	0.278818301					miR-942-5p	-1.48	0.027234629	0.159893627	miR-342-3p	-1.86	0.015757359	0.091957894
miR-20a-5p	0.84	0.049827511	0.287892284					miR-30c-2-3p	-4.32	0.028280615	0.163399106	miR-769-5p	-6.10	0.015915789	0.091957894
								miR-31-5p	-4.59	0.030192491	0.17171979	miR-150-5p	-1.68	0.01796157	0.100444116
								miR-877-5p	-1.19	0.031688023	0.175708731	miR-146b-3p	-7.42	0.018310872	0.100444116
								miR-224-5p	3.03	0.031859275	0.175708731	miR-204-5p	-7.05	0.018416506	0.100444116
								miR-889-3p	0.86	0.034648669	0.186175525	miR-638	-5.21	0.01848834	0.100444116
								miR-18a-3p	-3.67	0.034780043	0.186175525	let-7f-3p	-5.56	0.01932519	0.103313988
								miR-665	-1.15	0.039419278	0.207950974	miR-106b-5p	1.39	0.019584245	0.103313988
								miR-148b-3p	0.70	0.040171075	0.208889592	miR-24-3p	0.52	0.020504727	0.106624579
								let-7i-5p	0.45	0.041874986	0.214683029	miR-378a	-0.77	0.021308045	0.109241242
								miR-93-5p	0.69	0.043452277	0.219675401	miR-193a-5p	0.95	0.021772478	0.109844827
								miR-16-2-3p	-0.73	0.044755518	0.223164501	miR-99b-3p	-4.92	0.02202932	0.109844827
								miR-23a-3p	0.33	0.045759572	0.225087625	miR-7180-3p	-7.06	0.022375583	0.11006368
												miR-361-3p	-3.46	0.022940171	0.111336295
												miR-429-3p	-6.08	0.023867445	0.113231705
												miR-23a-3p	0.50	0.024138687	0.113231705
												miR-542-3p	-4.70	0.024453938	0.113231705
												miR-423-3p	-0.55	0.024575013	0.113231705
												miR-1284	-6.94	0.025963594	0.118134352
												miR-192-5p	-0.91	0.029146975	0.128489732
												miR-27a-3p	0.62	0.029268116	0.128489732
												miR-491-5p	-4.98	0.029646972	0.128489732
												miR-874-3p	-2.65	0.029918842	0.128489732
												miR-136	-6.63	0.03030399	0.128489732
												miR-30b-3p	-6.57	0.030357464	0.128489732
												miR-143-3p	0.93	0.030805587	0.128685122
												miR-320a	-0.76	0.031110689	0.128685122
												miR-484	-0.59	0.03274926	0.133940795

												miR-323a-5p	-5.75	0.033766134	0.135230942
												miR-421	-2.86	0.033807735	0.135230942
												miR-188-5p	-6.35	0.036515785	0.144475499
												miR-29b-3p	-2.38	0.040845459	0.157890093
												miR-889-3p	1.09	0.040910793	0.157890093
												miR-378d	-1.18	0.041207579	0.157890093
												let-7f-5p	-0.75	0.043302465	0.16146828
												miR-338-3p	-6.08	0.043369371	0.16146828
												miR-369-5p	-5.20	0.043472229	0.16146828
												miR-195-5p	-4.41	0.044667612	0.164232432
												miR-29b-2-5p	-4.87	0.046045118	0.167604229
												let-7i-5p	0.58	0.048860889	0.17583201
												miR-221-5p	1.74	0.049606806	0.17583201
												miR-330-3p	-1.93	0.049754662	0.17583201

Supplemental Table 3. TAM 2.0 Pathway Analysis and Enriched miRNAs

Note: TAM 2.0 backtraces mature miRNAs into potential pri-miRNAs. Pri-miRNAs are denoted by the addition of "-1", "-2", etc. after the miRNA number, but the pri-miRNA number can be discarded for our analysis

Aging	Apoptosis	Immune Response	Hematopoiesis	Tumor Supressor miRNAs
let-7a-2	miR-181a-1	miR-200c	let-7a-2	let-7a-2
let-7b	miR-92a-1	miR-181a-1	let-7b	let-7b
miR-7-1	miR-16-2	miR-92a-1	miR-486-2	miR-7-1
miR-200c	miR-7-2	miR-486-1	miR-181a-1	miR-200a
miR-7-3	miR-144	miR-16-2	miR-92a-1	miR-200c
miR-148a	miR-181a-2	miR-100	miR-486-1	miR-7-3
miR-181a-1	miR-7-1	miR-181a-2	let-7a-3	miR-181a-1
miR-92a-1	miR-7-3	miR-30a	miR-100	let-7a-3
let-7a-3	miR-30a	miR-92a-2	miR-378a	miR-16-2
miR-30a	miR-92a-2	miR-486-2	let-7a-1	miR-7-2
miR-16-2	miR-216b	miR-217	miR-144	let-7a-1
miR-100	miR-193a	miR-148a	miR-92a-2	miR-181a-2
miR-7-2	miR-148a		miR-181a-2	
let-7a-1	miR-216a			
miR-144				
miR-92a-2				
miR-181a-2				

Inflammation	Cell Death	Hormone-mediated Signaling Pathway	Epithelial-to-Mesenchymal Transition	Cell Cycle
miR-181a-1	let-7a-2	let-7a-2	let-7a-2	let-7a-2
miR-100	let-7b	miR-7-3	let-7b	let-7b
miR-7-2	miR-7-1	miR-92a-1	miR-215	miR-200c
miR-144	miR-7-3	let-7a-3	miR-486-2	miR-92a-1
miR-181a-2	miR-181a-1	miR-16-2	miR-200a	let-7a-3
miR-7-1	miR-92a-1	miR-7-2	miR-200c	miR-191
miR-7-3	let-7a-3	miR-375	miR-486-1	miR-16-2
miR-148a	miR-16-2	let-7a-1	let-7a-3	let-7a-1
	miR-7-2	miR-92a-2	miR-191	miR-92a-2
	let-7a-1	miR-7-1	miR-30a	
	miR-92a-2		miR-100	
	miR-181a-2		miR-375	
			let-7a-1	
			miR-144	

Angiogenesis	Adipocyte Differentiation	Regulation of Stem Cell	Cell Proliferation	Bone Regeneration
let-7b	let-7a-2	miR-200a	let-7a-2	let-7b
miR-486-2	let-7b	miR-200c	let-7b	miR-92a-1
miR-92a-1	miR-92a-1	miR-148a	miR-200a	miR-92a-2
miR-486-1	let-7a-3	miR-181a-1	miR-200c	
miR-30a	miR-378a	miR-144	miR-92a-1	
miR-16-2	miR-375	miR-181a-2	miR-16-2	
miR-378a	let-7a-1		miR-378a	
miR-92a-2	miR-92a-2		let-7a-1	
			miR-92a-2	

Supplemental Table 4. Modulated miRNAs from the serum of patients with viral/bacterial infection or inflammatory bowel disease

Note: Tables sources are listed in Discussion section. Different studies used different metric (i.e., Fold-change or △△Ct). HC = health control

HCV vs HC			Avian Influenza A (H7N9) vs HC			Pulmonary tuberculosis infection vs HC			Crohn's disease versus HC		
miRNA	Fold-Change HCV_1	Fold-Change HCV_3	miRNA	△△Ct	2−	miRNA	△△CT	2-△△CT	miRNA	log2 FC	P-value
miR-451	11.04	11.9	miR-1290	−10.05	1063.66	miR-210-4373089	-10.46	1412.63	miR-27a*	2·66	0·035
miR-21	8.38	11.51	miR-1275	−9.36	655.36	miR-432-4373280	-9.47	711.39	miR-140-3p	1·89	0·012
miR-19b	7.33	10.48	miR-1260	−9.25	609.09	miR-423-5p-4395451	-8.8	444.34	miR-140-5p	1·44	0·017
miR-16	7.53	10.46	miR-574-3p	−4.49	22.48	miR-134-4373299	-8.63	396.99	miR-16	1·43	0·020
miR-1246	8.13	10.13	miR-454	−4.45	21.93	miR-144*-4395259	-8.38	333.44	miR-195	1·33	0·035
miR-130a	7.64	10.01	miR-148a	−4.38	20.85	miR-335-4373045	-7.85	230.56	miR-877	−1·13	0·029
miR-24	0	9.91	miR-539	−4.36	20.57	miR-26a-4395166	-7.72	210.39	miR-532-5p	2·45	0·030
miR-27a	0	9.87	miR-223	−4.27	19.29	miR-130b-4373144	-7.67	203.73	miR-15b	2·27	0·031
miR-23a	0	9.79	miR-142-5p	−4.27	19.26	miR-26b-4395167	-7.61	194.83	miR-27a	2·04	0·026
miR-1290	8.13	9.75	miR-485-3p	−4.21	18.56	miR-139-3p-4395424	-7.6	193.58	let-7g	1·94	0·012
miR-29a	0	9.36	miR-548c-5p	−4.13	17.56	miR-29a-4395223	-7.55	187.49	miR-93	1·89	0·013
miR-342-3p	0	8.84	miR-17	−4.12	17.41	miR-376c-4395233	-7.55	187.42	let-7d	1·76	0·040
miR-107	6.34	8.8	miR-484	−4.10	17.17	miR-376a-4373026	-7.53	184.8	miR-598	1·74	0·006
miR-1299	8.09	8.78	miR-652	−4.09	17.01	miR-425*-4395413	-7.52	183.24	miR-142-5p	1·71	0·024
miR-146a	0	8.63	miR-660	−4.07	16.78	miR-636-4395199	-7.26	153.29	let-7e	1·60	0·037
miR-19a	0	8.52	miR-20b	−4.06	16.66	miR-299-3p-4373189	-7.1	137.19	miR-223	1·45	0·023
miR-29c	0	8.4	miR-511	−4.05	16.61	miR-628-5p-4395544	-6.56	94.5	miR-374b	1·40	0·021
miR-185	0	8.27	miR-26b	−4.01	16.15	miR-590-5p-4395176	-6.54	92.94	miR-19a	1·40	0·038
miR-15a	6.08	8.27	miR-210	−4.01	16.15	miR-185-4395382	-6.53	92.67	miR-345	1·39	0·015
miR-150	0	8.14	miR-489	−3.98	15.78	miR-99b*-4395307	-6.53	92.57	miR-199a-3p	1·38	0·027
miR-25	0	8.05	miR-22*	−3.98	15.74	miR-340-4395369	-6.53	92.15	miR-24	1·15	0·013
miR-140-3p	0	7.97	miR-15a*	−3.97	15.7	miR-744-4395435	-6.5	90.21	miR-30e	1·09	0·044
miR-1323	0	7.91	miR-106a	−3.92	15.15	miR-409-3p-4395443	-6.4	84.29	miR-29a	1·07	0·006
miR-1305	0	7.85	miR-331-5p	−3.91	15.04	miR-330-3p-4373047	-6.31	79.2	mmiR-28-3p	1·01	0·039
miR-1915	0	7.75	miR-194	−3.88	14.73	miR-328-4373049	-6.14	70.69	mmiR-150	−1·54	0·011
miR-142-5p	0	7.71	miR-139-5p	−3.85	14.38	miR-433-4373205	-5.72	52.76	**Ulcerative Colitis versus HC**		
bkv-miR-B1-5p	0	7.7	miR-193a-5p	−3.84	14.37	miR-660-4380925	-5.54	46.55	miRNA	log2 FC	P-value
miR-20a	0	7.69	miR-29a	−3.80	13.94	miR-532-3p-4395466	-5.53	46.33	miR-760	5·07	0·048
miR-516a-5p	0	7.67	miR-24	−3.75	13.43	miR-505*-4395198	-5.51	45.58	miR-423-5p	4·34	0·044
miR-629	0	7.54	miR-140-5p	−3.71	13.12	miR-26a-1*-4395554	-5.5	45.28	miR-128	4·25	0·036
miR-130b	5.87	7.54	miR-28-3p	−3.69	12.91	miR-323-3p-4395338	-5.5	45.13	miR-196b	4·17	0·034
miR-887	0	7.5	miR-151-3p	−3.67	12.73	miR-487b-4378102	-5.49	44.99	miR-103	4·01	0·029
let-7i	0	7.47	miR-192	−3.66	12.62	miR-26b*-4395555	-5.49	44.89	miR-221	3·68	0·047
miR-526b	5.82	7.37	miR-190b	−3.60	12.15	miR-454*-4395185	-5.49	44.83			
miR-205	0	7.36	miR-143	−3.59	12.04	miR-598-4395179	-5.49	44.8			
miR-520b	0	7.35	miR-425	−3.58	11.96	miR-361-5p-4373035	-5.48	44.59			
miR-1300_v13.0	0	7.21	miR-101	−3.55	11.7	let-7e-4395517	-5.48	44.5			
miR-15b	0	7.14	miR-146b-5p	−3.52	11.45	miR-886-5p-4395304	-5.47	44.25			
miR-625	0	7.1	miR-26a	−3.47	11.06	miR-99b-4373007	-5.44	43.49			
miR-126	0	7.05	miR-425*	−3.46	11.02	miR-409-3p-4395443	-5.17	35.93			
miR-1260	6.8	7	miR-25	−3.45	10.92	miR-889-4395313	-5.05	33.13			
miR-584	7.51	6.98	miR-186	−3.44	10.86	miR-494-4395476	-4.97	31.32			
miR-196a	0	6.96	miR-191	−3.42	10.72	miR-200c-4395411	-4.64	25			
miR-365	0	6.88	miR-9*	−3.42	10.72	miR-25-4373071	-4.55	23.38			
miR-564	5.34	6.81	miR-494	−3.41	10.65	miR-196b-4395326	-4.53	23.03			
miR-30e	0	6.77	miR-323-3p	−3.41	10.64	miR-576-3p-4395462	-4.51	22.78			
miR-423-5p	5.39	6.74	miR-34a	−3.27	9.65	miR-127-3p-4373147	-4.5	22.66			
miR-583	0	6.61	miR-125a-5p	−3.26	9.58	miR-15b-4373122	-4.42	21.39			
miR-106b	0	6.6	miR-152	−3.25	9.51	miR-410-4378093	-4.32	20.03			
miR-1469	0	6.59	miR-19b	−3.24	9.45	miR-20b-4373263	-4.27	19.35			
let-7g	0	6.46	miR-532-5p	−3.23	9.41	miR-199a-3p-4395415	-4.13	17.46			
miR-1303	0	6.45	miR-324-5p	−3.21	9.25	miR-106a-4395280	-3.92	15.17			
let-7d	0	6.44	miR-106b	−3.19	9.12	miR-126*-4373269	-3.8	13.9			
let-7b	0	6.43	miR-886-5p	−3.19	9.11	miR-93-4373302	-3.75	13.47			
miR-221	0	6.4	miR-744	−3.14	8.83	miR-345-4395297	-3.66	12.68			
miR-663b	0	6.35	miR-451	−3.13	8.75	miR-27a-4373287	-3.61	12.23			
miR-654-5p	0	6.28	miR-130a	−3.13	8.73	let-7d-4395394	-3.55	11.74			
miR-516b	0	6.25	miR-200c	−3.11	8.63	miR-139-5p-4395400	-3.54	11.67			
miR-27b	0	6.23	miR-625*	−3.11	8.61	let-7g-4395393	-3.2	9.21			
miR-101	0	6.23	miR-532-3p	−3.10	8.56	miR-146a-4373132	-3.12	8.67			
ebv-miR-BART7	3.49	6.22	miR-720	−3.08	8.44	miR-21-4373090	-3.06	8.31			
miR-425	0	6.12	miR-338-5p	−3.05	8.3	miR-20a-4373286	-2.9	7.46			
miR-17	0	6.08	miR-324-3p	−3.05	8.29	miR-24-4373072	-2.8	6.95			
miR-122	0	6.08	miR-203	−3.05	8.27	miR-133a-4395357	-2.78	6.87			
miR-492	0	6.04	miR-30a-3p	−3.01	8.07	miR-301a-4373064	-2.77	6.83			
miR-1470	0	5.98	miR-345	−3.01	8.04	miR-17-4395419	-2.62	6.14			
miR-361-5p	0	5.97	miR-27a	−2.92	7.57	miR-320-4395388	-2.59	6.03			
miR-302c	0	5.96	miR-30e-3p	−2.88	7.37	miR-223-4395406	-2.51	5.7			

miR-151-3p	0	5.94	miR-664	−2.86	7.28	miR-339-3p-4395295	-2.37	5.16			
miR-1208	6	5.94	miR-340	−2.77	6.84	miR-103-4373158	-2.35	5.09			
miR-148b	0	5.89	miR-495	−2.76	6.79	miR-28-3p-4395557	-2.34	5.08			
miR-196b	0	5.87	miR-21	−2.75	6.74	miR-574-3p-4395460	-2.33	5.01			
miR-199a-3p	0	5.83	miR-335	−2.72	6.58	miR-92a-4395169	-2.04	4.13			
miR-199a-5p	0	5.83	miR-181a	−2.71	6.53	miR-885-5p-4395407	-1.99	3.99			
miR-1182	4.51	5.71	miR-218	−2.62	6.16	miR-193b-4395478	-1.99	3.97			
miR-345	0	5.33	miR-223*	−2.58	5.99	miR-106b-4373155	-1.93	3.81			
miR-183	0	5.29	miR-93*	−2.57	5.95	miR-126-4395339	-1.9	3.74			
miR-186	0	5.28	miR-151-5p	−2.52	5.74	miR-30d-4373059	-1.87	3.67			
miR-30d	4.19	5.14	miR-27b	−2.51	5.7	miR-625*-4395543	-1.81	3.5			
miR-18b	0	4.88	miR-199a-5p	−2.47	5.56	miR-145-4395389	-1.81	3.49			
miR-520e	-2.41	4.79	miR-505	−2.47	5.53	miR-197-4373102	-1.73	3.33			
miR-1237	-3.32	4.61	miR-93	−2.38	5.21	miR-151-3p-4395365	-1.62	3.08			
miR-328	-2.74	4.55	miR-144*	−2.34	5.06	miR-30d-4373059	-1.53	2.89			
miR-125a-3p	-3.33	4.54	let-7g	−2.34	5.06	miR-125a-5p-4395309	-1.41	2.65			
miR-30c-2	3.15	4.38	miR-130b	−2.32	5	miR-140-5p-4373374	-1.38	2.6			
miR-373	3.28	4.27	miR-128	−2.23	4.7	miR-19a-4373099	-1.37	2.59			
hcmv-miR-US33-5p	-2.28	4.17	miR-132	−2.22	4.66	miR-30c-4373060	-1.32	2.5			
miR-223	0.53	3.99	miR-331-3p	−2.10	4.29	miR-425-4380926	-1.28	2.43			
miR-324-3p	-2.94	3.84	miR-375	−2.07	4.2	miR-19b-4373098	-1.05	2.07			
miR-769-3p	1.21	3.7	miR-125b	−2.02	4.06	miR-142-3p-4373136	-1.03	2.05			
miR-22	1.36	3.69	miR-148b	−2.00	4	miR-584-4381026	6.7	0.01			
miR-659	1.46	3.67	miR-19b-1*	−2.00	3.99	miR-485-3p-4378095	7.51	0.01			
miR-198	1.73	3.48	miR-122	−1.90	3.73	miR-768-3p-4395188	7.74	0			
miR-30c-1	1.68	3.48	miR-486-5p	−1.86	3.64	miR-610-4380980	8.46	0			
miR-665	-2.29	3.41	miR-409-3p	−1.85	3.6	miR-30b*-4395240	8.47	0			
miR-874	1.1	3.41	miR-142-3p	−1.81	3.5	miR-501-5p-4373226	9.48	0			
miR-622	-2.92	3.4	miR-150	−1.80	3.48	miR-571-4381016	11.71	0			
miR-320d	2.72	3.29	let-7b	−1.76	3.38						
miR-149	1.08	3.26	miR-541*	−1.73	3.32						
miR-320c	1.74	3.24	miR-221	−1.62	3.08						
miR-1275	1.63	3.21	miR-649	−1.61	3.06						
miR-765	1.4	3.16	miR-92a	−1.54	2.92						
miR-483-5p	1.25	3.06	miR-518f	−1.33	2.51						
miR-1229	-2.39	2.99	has-miR-155	−1.28	2.43						
miR-188-5p	0.8	2.98	miR-301a	−1.12	2.17						
miR-1202	0.89	2.87	miR-486-3p	−1.07	2.1						
miR-187	1.34	2.83	miR-10a	−1.05	2.07						
miR-1207-5p	0.83	2.78	miR-642	1.09	0.47						
miR-371-3p	-3.02	2.76	miR-654-3p	1.22	0.43						
miR-150	1.21	2.72	let-7e	1.28	0.41						
miR-1225-5p	0.71	2.71	miR-548c-3p	1.34	0.39						
miR-1268	1.23	2.69	miR-29b	1.41	0.38						
miR-134	1.02	2.63	miR-548p	1.77	0.29						
miR-623	1.24	2.51	miR-1243	9.65	0						
miR-1224-5p	1.27	2.51									
hcmv-miR-US4	0.85	2.39									
miR-1226	1.26	2.39									
miR-371-5p	1	2.38									
miR-1249	0.63	2.38									
miR-557	0.83	2.23									
miR-1471	1.24	2.18									
miR-1274b	1.61	2.14									
miR-601	0.63	2.14									
miR-320b	1.27	2.04									
miR-135a	0.85	2.03									
miR-498	1.28	2									
miR-33b	1.06	2									
miR-296-5p	0.32	1.98									
miR-1914	0.32	1.94									
miR-671-5p	1.38	1.92									
miR-877	1.17	1.92									
miR-1181	0.77	1.9									
miR-1228	0.45	1.78									
miR-139-3p	0.98	1.74									
miR-484	-0.15	1.68									
ebv-miR-BART13	0.09	1.5									
miR-1915	0.48	1.49									
hcmv-miR-UL70-3p	0.77	1.49									
miR-663	-0.66	1.46									
miR-638	0.39	1.45									

miR-630	0.61	1.39									
miR-149	-5.83	1.39									
miR-1234	0.27	1.29									
let-7f-1	-5.72	1.27									
miR-320a	0.26	1.22									
miR-1539	-0.55	1.18									
miR-197	-0.52	1.16									
miR-1225-3p	0.25	1.11									
miR-1183	-2.55	1.06									
miR-1238	0.18	1.06									
miR-720	1.57	0.99									
miR-191	0.24	0.99									
miR-575	0.28	0.91									
miR-550a	-1.06	0.91									
miR-486-5p	0.88	0.79									
miR-636	-4.47	0.74									
let-7b	-6.56	0.68									
miR-602	-6.53	0.64									
miR-766	-0.64	0.54									
miR-1281	0.43	0.42									
miR-1280	-0.03	0.24									
miR-425	-0.05	0.23									
miR-1825	0.3	0.1									
miR-1287	6.89	0									
miR-144	8.7	0									
miR-923_v12.0	-1.16	-0.04									
miR-877	-0.63	-0.1									
ebv-miR-BART12	1.88	-0.18									
miR-572	-0.77	-0.84									
miR-32	1.25	-1									
miR-563	2.56	-2.63									
miR-422a	-2.8	-2.8									
miR-494	-3.2	-3.2									
ebv-miR-BART16	3.08	-3.23									
miR-483-3p	0.47	-5.6									

Supplemental Table 5. Significantly modulated miRNAs between unirradiated NHPs and different treatment groups 96 hours post-irradiation NHPs.

Note: Siginifcantly modulated miRNAs are highlighted in red. Only miRNAs with a log2FC of (>.58) and a false discovery rate-adjujsted p-value of (<0.05) were considered significant.

Untreated vs Unirradiated				Ex-Rad I vs Unirridiated				Ex-Rad II vs Unirradiated			
miRNA	log2FC	PValue	FDR	miRNA	log2FC	PValue	FDR	miRNA	log2FC	PValue	FDR
miR-30a-5p	2.19	3.74E-16	1.11E-13	miR-30a-5p	2.46	3.10E-20	9.18E-18	miR-30a-5p	2.30	9.07E-18	2.68E-15
miR-21-5p	1.74	2.66E-11	3.94E-09	miR-21-5p	1.89	3.16E-13	4.68E-11	miR-143-3p	2.59	9.54E-14	1.41E-11
miR-26b-3p	-10.07	7.88E-07	6.58E-05	miR-27b-3p	1.47	9.04E-11	8.92E-09	miR-21-5p	1.73	3.80E-11	3.75E-09
miR-27b-3p	1.13	9.11E-07	6.58E-05	miR-101-3p	2.06	3.42E-08	2.53E-06	miR-10a-5p	1.47	3.57E-08	2.28E-06
miR-10a-5p	1.31	1.11E-06	6.58E-05	miR-143-3p	1.64	5.11E-06	0.000302397	miR-375	3.24	3.86E-08	2.28E-06
miR-101-3p	1.72	5.74E-06	0.0002831	miR-10a-5p	1.20	9.52E-06	0.000469871	miR-27b-3p	1.14	8.73E-07	4.31E-05
miR-142-5p	-1.24	1.96E-05	0.0008269	miR-144	1.54	3.51E-05	0.001485066	miR-200a-3p	3.85	1.48E-06	6.27E-05
miR-93-3p	-8.30	3.26E-05	0.0012034	miR-423-5p	-1.26	4.96E-05	0.001813299	miR-101-3p	1.69	8.69E-06	0.000321514
miR-200a-3p	3.31	3.66E-05	0.0012034	miR-27a-3p	1.02	5.51E-05	0.001813299	miR-10b-5p	1.24	5.08E-05	0.001672026
miR-197-3p	-4.78	6.74E-05	0.0019941	miR-30d-5p	1.03	0.000102483	0.003033504	miR-885-5p	-9.17	6.52E-05	0.001803785
miR-1296-5p	-8.79	9.26E-05	0.0024171	miR-615-3p	-8.41	0.000233059	0.006271404	miR-145-3p	2.27	6.70E-05	0.001803785
miR-885-5p	-9.17	9.80E-05	0.0024171	miR-10b-5p	1.12	0.000285834	0.007050567	miR-99a-5p	1.13	0.000177935	0.004389074
miR-381-3p	1.49	0.0001996	0.0045451	miR-6529-5p	-1.51	0.000455094	0.010362139	miR-484	-1.21	0.000240322	0.005471941
miR-342-5p	-8.92	0.0004999	0.0105701	miR-30e-5p	1.00	0.000684827	0.014479208	miR-340-3p	-8.37	0.000286351	0.006054275
miR-423-5p	-1.04	0.0006631	0.0112708	miR-942-3p	-8.44	0.000736381	0.01453125	miR-942-3p	-8.44	0.000522773	0.01031605
miR-92a-3p	-1.05	0.0006687	0.0112708	miR-200a-3p	2.71	0.000820272	0.015175025	miR-144	1.18	0.001817028	0.030654333
miR-340-5p	1.81	0.0007009	0.0112708	miR-375	1.93	0.001452814	0.025296048	miR-20a-5p	2.08	0.001845698	0.030654333
miR-486-5p	-1.21	0.0007047	0.0112708	miR-340-3p	-7.62	0.001737021	0.028564349	miR-125a-5p	0.88	0.001954882	0.030654333
miR-942-3p	-8.44	0.0007235	0.0112708	miR-381-3p	1.23	0.002354623	0.035737329	miR-185-3p	-8.01	0.001967677	0.030654333
miR-377-5p	-8.65	0.0013161	0.019153	miR-151-3p	1.08	0.002430567	0.035737329	miR-150-3p	-3.70	0.002749219	0.040688447
miR-590-3p	-7.41	0.0013588	0.019153	miR-21-3p	1.36	0.002535419	0.035737329	miR-7205-3p	-8.20	0.003035619	0.042787779
miR-17-3p	-8.86	0.0014415	0.0193953	miR-10b-3p	1.76	0.002831959	0.037165603	miR-769-5p	-7.21	0.004162906	0.056010014
miR-486-3p	-3.40	0.0019244	0.0247665	miR-92a-3p	-0.91	0.003039037	0.037165603	miR-23a-3p	0.71	0.004589842	0.056240945
miR-200c-3p	3.61	0.0021054	0.025967	miR-7180-5p	-8.16	0.0030899	0.037165603	miR-9-3-3p	-8.38	0.00475008	0.056240945
miR-30d-5p	0.82	0.0022932	0.0271521	miR-125a-5p	0.84	0.003138987	0.037165603	miR-9-3p	-8.38	0.00475008	0.056240945
miR-374a-5p	1.25	0.0025702	0.029261	miR-23a-3p	0.73	0.003629993	0.04019195	miR-423-5p	-0.82	0.006250323	0.071157521
miR-550-5p	-7.62	0.002847	0.0312112	miR-106b-5p	2.01	0.003735013	0.04019195	miR-654-3p	-1.14	0.006503082	0.071293045
miR-574	-3.76	0.0030297	0.0320285	miR-18a-3p	-7.65	0.003801941	0.04019195	miR-7204-3p	-7.45	0.007761324	0.082048281
miR-10b-5p	0.91	0.0035862	0.0366041	miR-181a-5p	0.83	0.00396671	0.040487795	miR-6529-5p	-1.10	0.008293775	0.0846537
miR-16-2-3p	-1.80	0.0039395	0.0388693	miR-532-3p	-8.38	0.005452463	0.053797635	miR-106b-5p	1.79	0.010122451	0.097921976
miR-7174-3p	-6.89	0.0041825	0.0399358	miR-376a-3p	-6.83	0.006271373	0.058399509	miR-412-5p	-7.07	0.010255342	0.097921976
miR-122a-5p	-1.64	0.0059118	0.0546838	miR-142-5p	-0.77	0.00631346	0.058399509	miR-30b-3p	-6.93	0.01091195	0.100935537
miR-10a-3p	2.46	0.0077184	0.0692315	miR-340-5p	1.41	0.009338525	0.081978068	miR-148a-3p	0.73	0.012607716	0.11308739
miR-339-5p	-2.39	0.0082311	0.0716586	miR-126	0.81	0.0094164	0.081978068	miR-379-3p	-6.89	0.013479537	0.117351261
miR-378a	-0.94	0.008993	0.0736805	miR-145-3p	1.50	0.010433707	0.085909412	miR-150-5p	-1.70	0.019952968	0.165606345
miR-149-5p	-7.55	0.0093077	0.0736805	miR-484	-0.82	0.010448442	0.085909412	miR-381-3p	0.96	0.020141312	0.165606345
miR-125b-2-3p	-3.20	0.00945	0.0736805	miR-221-5p	2.21	0.012929374	0.103132975	miR-222-3p	-1.25	0.023875739	0.189600925
miR-222-3p	-1.46	0.009459	0.0736805	miR-374a-5p	1.03	0.013859699	0.103132975	miR-340-5p	1.24	0.024340659	0.189600925
miR-1249	-6.92	0.0099921	0.0758371	miR-125b-1-3p	-6.60	0.013871389	0.103132975	miR-221-5p	2.00	0.025768716	0.194820771
miR-1306-5p	-7.87	0.0103095	0.0762899	miR-323b-3p	-1.47	0.014239495	0.103132975	miR-376a-3p	-5.77	0.026327131	0.194820771
miR-1224-5p	-6.05	0.0109837	0.0775439	miR-185-3p	-6.75	0.014285311	0.103132975	miR-30d-5p	0.60	0.027652883	0.199640328
miR-769-5p	-6.38	0.0110028	0.0775439	miR-320c	-5.05	0.015021053	0.103648292	miR-486-3p	-2.20	0.028984595	0.204272384
miR-139-3p	-6.55	0.0114331	0.0787021	miR-379-3p	-6.89	0.015057015	0.103648292	miR-665	-2.35	0.030232889	0.206408948
miR-429-3p	-6.51	0.0122982	0.0827336	miR-6827-3p	-7.03	0.019225249	0.129333493	let-7a-5p	-0.87	0.030730513	0.206408948
miR-30b-3p	-6.93	0.0133247	0.0858886	miR-24-3p	0.59	0.020526558	0.135019139	miR-186-5p	0.80	0.031379739	0.206408948
miR-150-5p	-1.82	0.0133475	0.0858886	miR-7174-3p	3.60	0.021545915	0.138643281	miR-323b-3p	-1.24	0.035184236	0.226402907
miR-148b-3p	1.31	0.0156329	0.0965192	miR-342-3p	-2.11	0.022427909	0.141248108	miR-320a	-0.75	0.036403229	0.229262887
miR-423-3p	-0.64	0.0160746	0.0965192	miR-25	0.65	0.025672023	0.154095513	miR-10a-3p	1.89	0.044213239	0.272648306
miR-136	-6.92	0.0165058	0.0965192	miR-320a	-0.80	0.025923854	0.154095513	miR-30d-3p	-5.40	0.047345577	0.276101747
miR-205	-4.07	0.0165898	0.0965192	miR-150-3p	-2.54	0.026029647	0.154095513	miR-140-5p	-5.77	0.047826291	0.276101747
miR-299-5p	-7.18	0.01663	0.0965192	miR-421	-4.13	0.029261299	0.168787283	miR-130b-5p	-3.95	0.048154572	0.276101747
miR-497-5p	-6.91	0.0176065	0.097656	miR-486-5p	-0.75	0.02965182	0.168787283	let-7f-5p	-0.72	0.048504361	0.276101747
miR-192-5p	-1.02	0.0176468	0.097656	miR-339-5p	-1.81	0.036486488	0.203773591				
miR-505-5p	-6.00	0.0179075	0.097656	miR-16-2-3p	-1.25	0.037364607	0.204813403				
miR-320a	-0.85	0.0181455	0.097656	miR-148a-3p	0.61	0.039855973	0.214497603				
miR-1-3p	2.61	0.019001	0.0980909	miR-30c-5p	-0.83	0.044592906	0.235705363				
miR-151-3p	0.85	0.0190164	0.0980909	miR-30b-5p	-1.91	0.047691722	0.246020754				
miR-10b-3p	1.40	0.0192205	0.0980909	let-7f-5p	-0.72	0.04838215	0.246020754				
let-7f-5p	-0.84	0.023	0.1148467	miR-193b-5p	-3.40	0.049474993	0.246020754				
miR-375	1.41	0.0233372	0.1148467	miR-150-5p	-1.40	0.049869072	0.246020754				
miR-204-5p	-7.47	0.0236677	0.1148467								
miR-99b-3p	-5.31	0.0253943	0.1208318								
miR-92b-3p	-1.52	0.0257176	0.1208318								
miR-214-3p	-2.64	0.0266973	0.1216086								
miR-143-3p	0.83	0.0269823	0.1216086								
miR-22	0.57	0.0271154	0.1216086								
miR-410-3p	1.27	0.0282978	0.1250172								
miR-26a-5p	0.54	0.0315908	0.1375127								
miR-484	-0.68	0.0330413	0.1417423								
miR-342-3p	-1.89	0.0377703	0.1579967								
miR-296-3p	-2.57	0.0378979	0.1579967								

miR-369-5p	-5.52	0.0416093	0.1681418								
miR-27a-3p	0.53	0.0418584	0.1681418								
miR-874-3p	-2.88	0.0420354	0.1681418								
miR-6529-5p	-0.81	0.046342	0.1828966								
miR-361-3p	-3.50	0.0477516	0.1859797								

Supplemental Table 6. Significantly modulated miRNAs between untreated and Ex-Rad treated NHPs 96 hours post-irradiation

Untreated vs Ex-Rad I				Untreated vs Ex-Rad II			
miRNA	log2FC	PValue	FDR	miRNA	log2FC	PValue	FDR
miR-7174-3p	10.88	3.00E-05	0.003334171	miR-26b-3p	9.75	4.23E-05	0.009383755
miR-26b-3p	9.69	4.50E-05	0.003334171	miR-93-3p	8.45	0.0001808	0.016567705
miR-144	1.95	4.51E-05	0.003334171	miR-144	1.76	0.0002239	0.016567705
miR-93-3p	7.44	0.000588012	0.032634681	miR-7174-3p	8.07	0.0004574	0.025386717
miR-197-3p	4.80	0.001770547	0.078612296	miR-143-3p	1.62	0.0018665	0.08287444
miR-16-5p	1.12	0.024903431	0.849654247	miR-122a-5p	2.14	0.002715	0.094646695
miR-125b-2-3p	3.32	0.030842092	0.849654247	miR-197-3p	4.49	0.0029844	0.094646695
miR-134-5p	-1.34	0.032769699	0.849654247	miR-486-5p	1.02	0.0054382	0.15091037
miR-126	0.89	0.035702094	0.849654247	miR-125b-2-3p	4.36	0.0069272	0.170870157
miR-200c-3p	-3.26	0.038272714	0.849654247	miR-375	1.73	0.0164059	0.334202616
miR-574	3.12	0.046065738	0.929690357	miR-370-3p	-1.29	0.0165596	0.334202616
				miR-451	1.19	0.0182704	0.338001722
				miR-145-3p	1.82	0.0331008	0.565259678
				miR-20a-5p	1.79	0.048653	0.650744469
				miR-574	3.06	0.0493385	0.650744469
				miR-99a-5p	0.74	0.0497736	0.650744469
